# Strong and efficient bismuth telluride-based thermoelectrics for Peltier microcoolers

**DOI:** 10.1093/nsr/nwae329

**Published:** 2024-09-16

**Authors:** Hua-Lu Zhuang, Bowen Cai, Yu Pan, Bin Su, Yilin Jiang, Jun Pei, Fengming Liu, Haihua Hu, Jincheng Yu, Jing-Wei Li, Zhengqin Wang, Zhanran Han, Hezhang Li, Chao Wang, Jing-Feng Li

**Affiliations:** State Key Laboratory of New Ceramics and Fine Processing, School of Materials Science and Engineering, Tsinghua University, Beijing 100084, China; Guangxi Pilot Free Trade Zone Jianju Technology Co., LTD., Qinzhou 535000, China; Max Planck Institute for Chemical Physics of Solids, Dresden 01187, Germany; State Key Laboratory of New Ceramics and Fine Processing, School of Materials Science and Engineering, Tsinghua University, Beijing 100084, China; State Key Laboratory of New Ceramics and Fine Processing, School of Materials Science and Engineering, Tsinghua University, Beijing 100084, China; State Key Laboratory of New Ceramics and Fine Processing, School of Materials Science and Engineering, Tsinghua University, Beijing 100084, China; Guangxi Pilot Free Trade Zone Jianju Technology Co., LTD., Qinzhou 535000, China; State Key Laboratory of New Ceramics and Fine Processing, School of Materials Science and Engineering, Tsinghua University, Beijing 100084, China; State Key Laboratory of New Ceramics and Fine Processing, School of Materials Science and Engineering, Tsinghua University, Beijing 100084, China; State Key Laboratory of New Ceramics and Fine Processing, School of Materials Science and Engineering, Tsinghua University, Beijing 100084, China; State Key Laboratory of New Ceramics and Fine Processing, School of Materials Science and Engineering, Tsinghua University, Beijing 100084, China; State Key Laboratory of New Ceramics and Fine Processing, School of Materials Science and Engineering, Tsinghua University, Beijing 100084, China; State Key Laboratory of New Ceramics and Fine Processing, School of Materials Science and Engineering, Tsinghua University, Beijing 100084, China; Department of Precision Instrument, Tsinghua University, Beijing 100084, China; Department of Precision Instrument, Tsinghua University, Beijing 100084, China; State Key Laboratory of New Ceramics and Fine Processing, School of Materials Science and Engineering, Tsinghua University, Beijing 100084, China

**Keywords:** bismuth telluride, thermoelectric, powder metallurgy, precision processing, Peltier cooler, microdevice

## Abstract

Thermoelectric Peltier coolers (PCs) are being increasingly used as temperature stabilizers for optoelectronic devices. Increasing integration drives PC miniaturization, requiring thermoelectric materials with good strength. We demonstrate a simultaneous gain of thermoelectric and mechanical performance in (Bi, Sb)_2_Te_3_, and successfully fabricate micro PCs (2 × 2 mm^2^ cross-section) that show excellent maximum cooling temperature difference of 89.3 K with a hot-side temperature of 348 K. A multi-step process involving annealing, hot-forging and composition design, is developed to modify the atomic defects and nano- and microstructures. The peak *ZT* is improved to ∼1.50 at 348 K, and the flexural and compressive strengths are significantly enhanced to ∼140 MPa and ∼224 MPa, respectively. These achievements hold great potential for advancing solid-state refrigeration technology in small spaces.

## Teaser

Superior mechanical and thermoelectric performance is simultaneously achieved in the (Bi, Sb)_2_Te_3_ nanocomposite through an innovative synthesis process, enabling the fabrication of efficient Peltier microcoolers.

## INTRODUCTION

Peltier coolers (PCs) are indispensable components of 5G optical modules [1], providing precise temperature management without any mechanical moving parts, thereby guaranteeing stable signal transport. The rapid development of 5G communication has generated a significant market demand for PCs. Currently, only one type of PC, namely the traditional π-type PC based on Bi_2_Te_3_-based alloys, can be utilized in this application due to its excellent cooling performance [2,3]. This performance stems from two aspects [2,3]: (i) efficient heat transfer perpendicular to the heating surface in the three-dimensional (3D) structure of π-type PCs, which is superior to devices with a two-dimensional (2D) structure due to their parasitic heat loss through the substrate; (ii) unparalleled thermoelectric (TE) performance of Bi_2_Te_3_-based alloys at near room temperature. However, with increasing integration and power density of 5G modules, such PCs face significant challenges: they need to become smaller with higher packing density that requires smaller TE legs while maintaining high TE performance for efficient cooling [3]. To meet the demands of large-scale industrial production, Bi_2_Te_3_-based alloys must have high mechanical properties to achieve excellent machinability. Unfortunately, commercially available Bi_2_Te_3_-based alloys typically prepared by zone-melting have poor processability owing to weak mechanical properties, which are easily cleaved because of the van der Waals bonding between Te(1)-Te(1) layers within their quasi-layered crystalline structure [4]. Therefore, it is necessary to improve the mechanical properties of Bi_2_Te_3_-based alloys without degrading TE performance to enable the fabrication of micro PCs with high cooling performance.

In recent years, powder metallurgy has gained widespread recognition for preparing TE materials with enhanced mechanical properties through grain refinement [5,6]. Simultaneously, this technique can improve the TE performance of Bi_2_Te_3_-based alloys by reducing the lattice thermal conductivity (*κ*_L_) through the introduction of atomic point defects, dislocations, grain boundaries and nano distortions [5]. Moreover, powder metallurgy technology enables easy fabrication of nanocomposites by directly incorporating additives as raw materials, which can further enhance both mechanical and TE properties [7]. Consequently, powder metallurgically fabricated materials have been increasingly used to produce smaller PCs. However, current reports indicate that the mechanical properties of Bi_2_Te_3_-based alloys prepared using powder metallurgy are still insufficiently developed, confining the processing sizes to a few hundred micrometers [8,9]. This poses challenges for scaling up the processing of TE legs on the order of several tens of micrometers, for which adding nanoscale secondary phases to further enhance processability is of great interest.

Bi_2_Te_3_-based alloys and their nanocomposites can be effectively synthesized through simple mechanical alloying (MA) of raw materials and subsequent spark plasma sintering (SPS), making this straightforward process suitable for industrial production. Further annealing treatment has been proven to effectively enhance the composition uniformity of MA-SPSed samples in numerous reports [10–12]. Nevertheless, the annealing process causes expansion of intrinsic micropores, resulting in volumetric expansion and a reduction in density that adversely affects their mechanical properties [13,14], for which we propose that further processing (e.g. hot forging) is necessary for obtaining a uniform bulk with high density. Moreover, re-closing of the expanded pores is expected to reduce the number of micropores, thereby leading to reduced stress concentration around these micropores during loading and hence enhanced mechanical properties.

**Figure 1.**
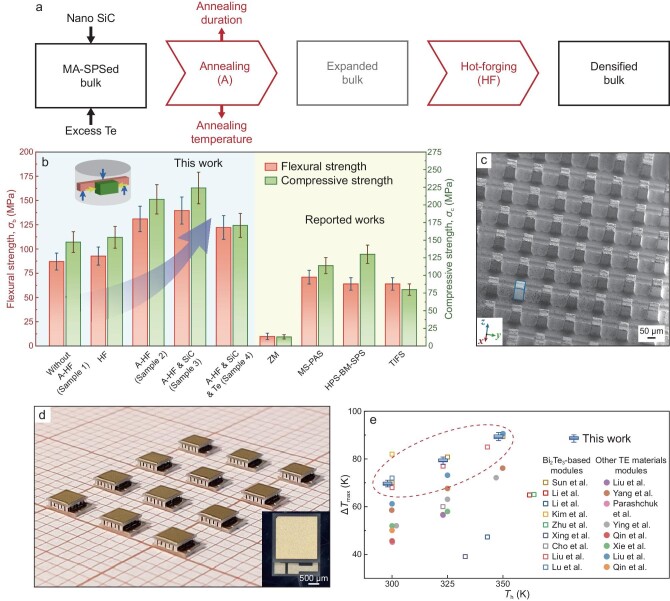
The multi-step process to obtain (Bi, Sb)_2_Te_3_ with high processability, as well as the fabricated high-performance micro PCs. (a) The flow chart of the multi-step process involving annealing and hot-forging. (b) The flexural and compressive strength of (Bi, Sb)_2_Te_3_ in this work at different stages, or in some representative publications [20–22]. (c) The micro cuboid pillar arrays on the surface of Sample 4, produced using a dicing saw. (d) A photo of the prepared micro PCs. (e) Comparison of the maximum cooling temperature of our micro PC with those in other works [23–39].

We exploited a fabrication process that synergistically enhances the mechanical and TE properties of Bi_2_Te_3_-based alloys by engineering atomic point defects and nano-/micro-structures, enabling the microfabrication of high-performance PCs. The designed process flow chart is depicted in Fig. 1a. (Bi, Sb)_2_Te_3_ bulks were initially fabricated by MA and SPS, and then annealed to control their composition uniformity with a subsequent hot forging process. Notably, hot forging not only increases the density but also modifies the microstructures by reducing micropores and facilitating defect evolution, resulting in superior mechanical properties and improved TE performance. The detailed schematic diagram is illustrated in Fig. S1, and the fabrication process involving annealing and hot forging is abbreviated to A-HF process. Following this A-HF process, a (Bi, Sb)_2_Te_3_ nanocomposite containing nano SiC and excess Te presents a peak *ZT* of ∼1.50 with excellent processability. SiC is added to introduce a dispersion strengthening effect [15–17], and excess Te is adopted to reduce the short range defects [18]. Subsequently, high-performance micro PCs with a cross-section area of 2 × 2 mm^2^ were successfully fabricated, which show exceptional cooling performance with a maximum cooling temperature difference (Δ*T*_max_) of 89.3 K when the hot-side temperature (*T*_h_) is 348 K.

## RESULTS AND DISCUSSION

### High mechanical performance and efficient micro PCs

The A-HF processed (Bi, Sb)_2_Te_3_ demonstrates significantly enhanced mechanical properties, making it suitable for the fabrication of micro PCs. The improvement in mechanical properties is evident from the average flexural and compressive strength measurements obtained through 3-point bending and compression tests (Fig. 1b; the fractured appearance of some specimens is shown in Fig. S2). The mechanical performance of different samples fabricated by the stepwise optimized fabrication process is investigated. Here, the sample fabricated with MA-SPS without A-HF, MA-SPS with A-HF, MA-SPS with A-HF and SiC inclusion, and MA-SPS with A-HF and SiC/excess Te are defined as Sample 1–4, respectively. Compared to Sample 1, Sample 2 shows a 50% increase in flexural strength from ∼87 MPa to ∼131 MPa and a 40% increase in compressive strength from ∼147 MPa to ∼207 MPa. It is noteworthy that, without the annealing process in advance, the HF process can only slightly increase the mechanical strength, suggesting the importance of annealing. The improved mechanical properties can be attributed to the evolutionary microstructures reflected by reduced micropores as well as the generated heterogeneous microstructures, which will be discussed later. Furthermore, the flexural and compressive strength can be maximized up to ∼140 MPa and ∼224 MPa, respectively, after further incorporating 0.4 vol% SiC nano-particles (Sample 3), benefiting from the dispersion strengthening effect of nanocomposites [15–17]. Notably, commercial (Bi, Sb)_2_Te_3_ prepared by zone-melting methods typically exhibit flexural and compressive strength of only ∼10 MPa. By further adding excess Te (Sample 4), which helps to improve TE performance [9], the mechanical properties are slightly reduced compared to the maximum value of Sample 3. This reduction may be attributed to the trace amount of Te distributing along the grain boundaries, which potentially facilitates grain boundary slip [19]. Nevertheless, these values are still higher than most of the reported values [20–22].

The high mechanical performance of Sample 4 ensures excellent processability for micro PCs. Small pieces of the strong Sample 4 were sliced using a dicing saw to produce micro cuboid pillar arrays on their surfaces, and the pillars serve as the legs of micro PCs. The size of these pillars reflects the processability of the material. Figure 1c demonstrates the diced pillar arrays on Sample 4, consisting of an array with 81 pillars each of them ∼50 × 50 × 120 μm^3^. Smaller pillars of ∼30 × 30 × 120 μm^3^ can be diced on Sample 3 (Fig. S3), i.e. A-HF processed (Bi, Sb)_2_Te_3_-SiC nanocomposites, which possess the highest mechanical properties in this work. Noteworthy, the cross-section area of ∼30 × 30 μm^2^ for TE legs represents the smallest value achieved through direct cutting using a dicing saw reported for Bi_2_Te_3_-based TE materials. Based on this small processable size, using Sample 4 as p-type legs and commercial n-type Bi_2_Te_3_ (TE performance depicted in Fig. S4) as n-type legs, large-scale manufacturing of micro PCs with a cross-section area of 2 × 2 mm^2^ containing 18 couples can be achieved (Fig. 1d).

The cooling performance of the micro PCs was subsequently tested. The alternating current (AC) internal resistance of the prepared micro PCs fluctuates less in the low resistance range (Fig. S5a). Notably, Δ*T*_max_ reaches 69.6 K, 79.3 K and 89.3 K with *T*_h_ at 298 K, 323 K and 348 K (Fig. 1e), respectively. The Δ*T*_max_ is stable with the test current ranging from 7 mA to 13 mA (Fig. S5b) and is competitive with reported PCs with much larger sizes (20 × 20 × 4.7 mm^3^) [23]. Moreover, this Δ*T*_max_ surpasses most results reported by other studies in TE materials not limited to Bi_2_Te_3_-based alloys [23–39]. In addition, the cooling coefficient of performance (COP) of the micro PCs at *T*_h_ = 325 K was measured, reaching 6.6 with a temperature difference (Δ*T*) of 0 K when the current is 0.1 A (Fig. S6).

### Improved thermoelectric performance

**Figure 2.**
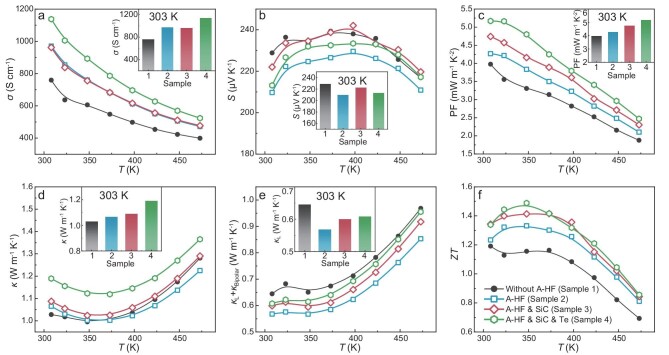
The TE performance of (Bi, Sb)_2_Te_3_ samples via different fabrication routes. Temperature dependence of (a) electrical conductivity, (b) Seebeck coefficient, (c) power factor, (d) total thermal conductivity, (e) lattice and bipolar thermal conductivity, and (f) *ZT* value for (Bi, Sb)_2_Te_3_ samples under different fabrication processes. The insets show the values at 303 K.

The high cooling performance of the micro PCs originates from their good TE performance. Figure 2 illustrates the overall enhancement in TE performance from Sample 1 to Sample 4. Detailed investigations of the A-HF process parameters and sample composition can be found in the Supplementary Data (Figs S7–S18). The electrical conductivity (*σ*) generally increases after each step of fabrication process optimization (Fig. 2a), rising from 759 to 1138 S·cm^−1^ from Sample 1 to Sample 4 at 303 K. In contrast, the Seebeck coefficient (*S*) does not exhibit a monotonic trend. Despite the different variation trends for *σ* and *S*, there is a consistent increase in power factor (PF) over the entire temperature range by each step. Notably, PF experiences a significant increase of ∼58% from 3.3 to 5.2 mW·m^−1^·K^−2^ at 303 K after the 4 steps of fabrication process optimization.

The total thermal conductivity (*κ*) gradually increases due to the enhanced electronic thermal conductivity (Fig. 2d), and the *κ*_L_ generally decreases after the 4 steps of fabrication process optimization (Fig. 2e). The A-HF process plays an important role in the reduction of *κ*_L_ from 0.65 to 0.57 W·m^−1^·K^−1^ from Sample 1 to Sample 2 at 303 K. The decreased *κ*_L_ suggests enhanced phonon scattering, which is probably due to the generation of dislocations during the HF process [40]. However, the following Samples 3 and 4 demonstrate slightly increased *κ*_L_, which may be induced by the high thermal conductivity of SiC, and the weakened phonon scattering with the reduction of grain boundaries and ${{{\mathrm{V}^{^{..}}}}_{\mathrm{Te}}}$ in Sample 4. Finally, the *κ*_L_ for Sample 4 is ∼0.61 W·m^−1^·K^−1^, which is still lower than that of the pristine Sample 1. As a result, the *ZT* value is gradually improved over the entire temperature range due to simultaneously modulated electrical and thermal transport properties (Fig. 2f). Specifically, the peak *ZT* value increases from 1.20 at 303 K for Sample 1 to 1.34 at 348 K for Sample 2 by using the A-HF method, and further to 1.42 at 348 K for Sample 3 by incorporating nano SiC. Through the introduction of excess Te, a high peak *ZT* value of 1.50 is achieved at 348 K for Sample 4.

**Figure 3.**
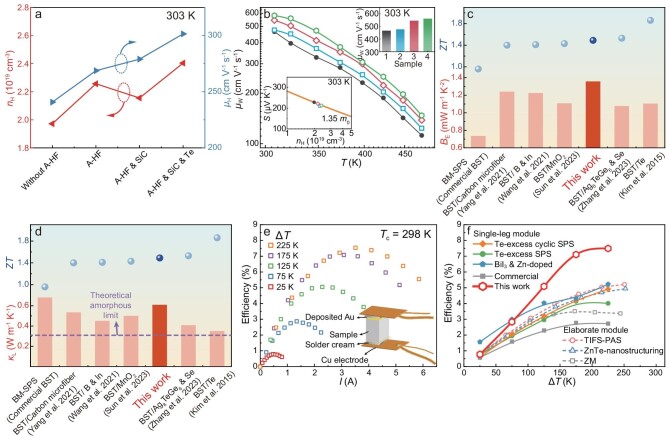
Electrical and thermal transport analysis for enhanced thermoelectric performance and energy conversion efficiency of the single-leg module. (a) The effects of fabrication processes on Hall carrier concentration and mobility at 303 K. (b) Temperature dependence of weighted mobility for samples with different fabrication processes (inset shows the values at 303 K and the Pisarenko curve at 303 K denoting m* = 1.35 *m*_0_). Comparison of (c) lattice thermal conductivity and (d) electronic quality factor in this work with other notable works in recent years [6,8,23,26,45,46]. *ZT* values are shown simultaneously. (e) Electrical current dependence of the measured TE energy conversion efficiency for the single-leg device fabricated using Sample 4. The inset schematics are the measuring setup. (f) The measured conversion efficiency as a function of temperature difference, in comparison with other measured values reported in recent years [19,21,47–50].

The enhanced electrical properties are further analyzed in detail with Hall measurements. It is found that the A-HF process enhances both Hall carrier concentration (*n*_H_) and mobility (*μ*_H_) from Sample 1 to Sample 2 (Fig. 3a), which benefits *σ*. The increase in *n*_H_ can be attributed to the increasing number of antisite defects (${\mathrm{S}}{{{\mathrm{b^{\prime}}}}_{{\mathrm{Te}}}}$ and ${\mathrm{B}}{{{\mathrm{i^{\prime}}}}_{{\mathrm{Te}}}}$) during annealing, which have lower formation energy compared to other defects [41]. The increased *n*_H_ also contributes to the decline in *S*. However, the increased antisite defect can also enhance carrier scattering. Therefore, there must be another reason for the simultaneous increase in *μ*_H_. Although there are no significant changes to the average grain size (Fig. S10), it is speculated that some nanograins may merge into larger grains during annealing, thereby weakening the grain boundary scattering. Further incorporation of nano SiC for Sample 3 slightly decreases *n*_H_ but improves *μ*_H_, resulting in very slight variations in *σ*, but an obvious increase in *S*, thereby further enhancement in PF. The incorporated nano SiC can amplify the donor-like effect by reinforcing the grinding effects during ball milling [42], thereby leading to decreased *n*_H_. As defects diminish due to this donor-like effect, the scattering of charge carriers caused by defects is reduced as well. Additionally, the decreased *n*_H_ can result in an increase in *μ*_H_. Therefore, despite potentially enhanced second phase scattering effects, *μ*_H_ increases for Sample 3. Furthermore, the *n*_H_ and *μ*_H_ are simultaneously increased for Sample 4 by adding excess Te, probably due to the reduction of ${{{\mathrm{V}^{^{..}}}}_{\mathrm{Te}}}$. Also, the slight reduction in grain boundaries leads to decreased dangling bonds (Fig. S16), which is equivalent to the reduced ${{{\mathrm{V}^{^{..}}}}_{\mathrm{Te}}}$ content [43]. There are no obvious changes in the effective mass, as does the band structure. The information about energy band structure can be inferred from the Pisarenko curve (see the inset in Fig. 3b). The above-mentioned improvements are based on optimizing defects and microstructures. Besides optimized carrier concentration, improvements primarily manifest through charge carrier transport characteristics as revealed by weight mobility values (*μ*_W_) (Fig. 3b).

The TE quality factor (*B*), consisting of the electronic quality factor (*B*_E_) and *κ*_L_, *B* = *B*_E_*T*/*κ*_L_, provides a guide for optimizing TE performance [44]. By comparing the *B*_E_ and *κ*_L_ of our Sample 4 to other notable works reported in recent years (Fig. 3c and d) [6,8,23,26,45,46], it is found that Sample 4 prepared using the optimized fabrication step in the present work exhibits greater potential for further enhancement in TE performance. First, the optimized Sample 4 demonstrates the highest *B*_E_ at 303 K compared to all listed samples, indicating a superior potential for optimizing carrier concentration towards a higher PF [44]. Second, *κ*_L_ at 303 K in this sample also surpasses that of most listed samples except for BM-SPSed commercial (Bi, Sb)_2_Te_3_, suggesting that there is greater room to further reduce *κ*_L_ by introducing more phonon scattering centers in our sample, which demonstrates the higher potential of our sample to further improve *ZT* by reducing *κ*_L_.

The energy conversion efficiency (*η*) of a single-leg device made from Sample 4 is investigated. With fixed cold side temperature (*T*_c_) at 298 K, the single-leg device performs increased *η* with rising Δ*T*, as shown in Fig. 3e (the output voltage, output power and heat flow are presented in Fig. S19). A maximum *η* of ∼7.5% is realized at Δ*T* = 225 K, which is higher than most reports involving Bi_2_Te_3_-based materials (Fig. 3f) [19,21,47–50]. Nevertheless, the experimental *η* values at each Δ*T* are still lower than the theoretically predicted *η* (Fig. S20a), indicating that there is still room to optimize the fabrication process and device structure. Specifically, through the comparison with simulated results in the Supplementary Data (Figs S19 and S20), it can be concluded that emphasis should be placed on improving diffusion barrier layers and soldering processes to reduce both the interface thermal and electric resistance while securing low heat flow between hot and cold sides.

### Evolutionary microstructure

**Figure 4.**
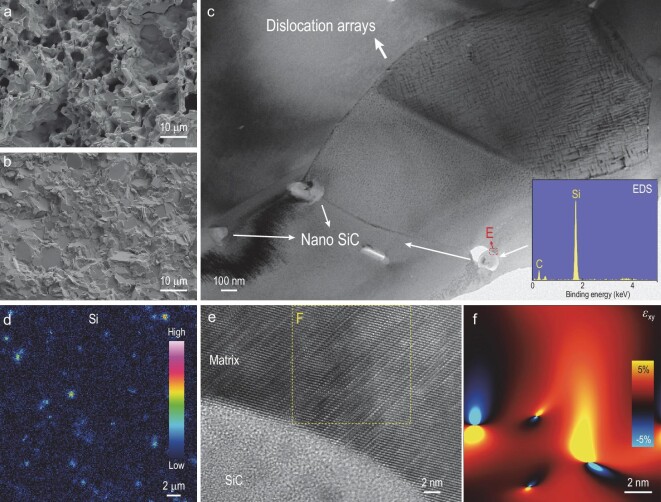
Microstructural characterization for Sample 4. SEM images of fracture surfaces for Sample 4 (a) before and (b) after HF process. (c) Low-magnification TEM image for Sample 4. (d) EPMA mapping for Si element on the polished surface of Sample 4. (e) High-resolution TEM image of a representative area showing the interface between nano SiC and matrix (area E in (c)). (f) The GPA corresponding to the matrix crystal lattice adjoining the nano SiC particle (area F in (e)).

The enhanced TE and mechanical properties originate essentially from the evolutionary microstructure due to the multiple fabrication steps undertaken in this work. The A-HF process does ensure the composition uniformity of the sample (Fig. S21), but more importantly, it makes the sample denser (Table S1). Scanning electron microscopy (SEM) analysis reveals that densification is linked to the reduction of micropores (Fig. S22). Notably, when solely subjected to HF without annealing, the micropores increase, leading to even lower density. SEM images of fracture surfaces reveal abundant interconnected large pores formed after annealing (Fig. 4a and Fig. S23), making the sintered bulks expand (Fig. S8). Similar phenomena in bismuth telluride have been reported by several works [13,14]. The subsequent HF process closes these interconnected pores, leading to sample densification (Fig. 4b). Consequently, the stress concentration around the micropores during loading can be alleviated, thereby enhancing their mechanical properties.

In addition, heterogeneous microstructures are induced by the HF process. Dislocation lines can be easily observed in Sample 4 by transmission electron microscopy (TEM), which exhibits various morphologies, represented by dislocation arrays (Fig. 4c). Moreover, some dislocations also manifest as dislocation networks, disordered dislocation lines and dislocation outcrops (Fig. S24). The distribution of these dislocations is nonuniform, primarily within the grains. Considering that the annealing process usually reduces dislocation, these dislocations probably result from significant grain deformation during the HF process [40]. These neatly arranged dislocations can hinder each other's motion, thereby being advantageous for better mechanical performance. Moreover, the high dislocation density can also enhance the mid-frequency phonon scattering, leading to the reduction of *κ*_L_ [51].

The incorporated nano SiC can be clearly observed (Fig. 4c and Fig. S25). In general, nano SiC is uniformly distributed throughout the sample, as detected by elemental mapping using an electronic probe microanalyzer (EPMA) (Fig. 4d). Different sizes of nano SiC are observed, indicative of SiC nanoparticle aggregation during sample preparation since the raw added SiC nanoparticles were approximately similar in size (Fig. S26). The presence of different-sized nano SiC particles serve as significant obstacles to dislocation motion, which further contributes to enhanced mechanical performance. The interface between an embedded nano SiC and the matrix was investigated using high-resolution TEM (HRTEM). However, it is challenging to observe the atomic structure of the SiC when observing the atomic structure of the matrix (Fig. 4e), which is probably due to the mismatched orientation relationship between them. Nevertheless, the incorporated nano SiC can induce additional strains into the matrix near the interface. Geometric phase analysis (GPA) illustrates both normal strains (*ε*_xx_ and *ε*_yy_) and shear strain (*ε*_xy_) within area F (Fig. 4f and Fig. S27), which reflect changes in unit cell volume and the deformation, respectively [52]. The magnitude of strains near the interface is greater than that away from it, highlighting contributions from the incorporated nano SiC. These strains probably result from lattice mismatch at the phase interface, which simultaneously lead to dislocations and enhanced deformation of the unit cell. Strain fluctuations in the matrix play an important role in enhancing phonon scattering, leading to the reduction of *κ*_L_. From all microstructure investigations, we highlight the significance of the proposed A-HF process and the composition design by SiC and Te incorporation, yielding strong high–TE-performance samples and hence the fabrication of efficient micro PCs with a small cross-section area of only 2 × 2 mm^2^.

## CONCLUSION

In summary, we successfully realized large-scale manufacturing of high-performance micro PCs with a cross-section of 2 × 2 mm^2^ using (Bi, Sb)_2_Te_3_ with improved mechanical and TE performance. The fabricated micro PCs demonstrated excellent cooling capabilities in an extremely compact size, showing Δ*T*_max_ of 69.6 K, 79.3 K and 89.3 K with *T*_h_ at 298 K, 323 K and 348 K, respectively. A flexural of ∼140 MPa, compressive strength of ∼224 MPa and a high maximum *ZT* value of ∼1.50 at 348 K are achieved. This high performance was attributed to the microstructure evolution resulting from the A-HF process. Fewer micropores, dense dislocations with various morphologies, and the nano SiC secondary phase are the main reasons for the enhanced mechanical strength and lower *κ*_L_, while fewer grain boundaries and ${{{\mathrm{V}^{^{..}}}}_{\mathrm{Te}}}$ are essential for the PF enhancement. This approach for improving (Bi, Sb)_2_Te_3_ enables the fabrication of high-performance micro PCs, which has great potential in the solid-state refrigeration industry.

## MATERIALS AND METHODS

### Synthesis

The starting materials of Bi, Sb and Te simple substance and SiC powder were first mixed and initially reacted via MA process according to the stoichiometric ratio of Bi_0.4_Sb_1.6_Te_3_, Bi_0.4_Sb_1.6_Te_3_–0.4 vol% SiC, and Bi_0.4_Sb_1.6_Te_3.01_–0.4 vol% SiC. The SiC content of 0.4 vol% was determined according to the optimal volume fraction reported in our previous study [15,42]. The MAed powders were then poured into graphite dies of 12 mm diameter and sintered by SPS. The MA-SPSed bulks were sealed into quartz tubes with a vacuum inside and placed in a muffle oven for annealing. Finally, the annealed bulks were loaded into a graphite die of a larger size (15 mm diameter) for the HF process by SPS. The process of SPS, annealing and HF are schematically shown in Fig. S1. The micro cuboid pillar arrays were processed using a dicing saw. The micro PCs were fabricated by Jianju Technology Co., Ltd, and the detailed fabrication process can be found in the Supplementary Data (Figs S28–S30).

### Structural characterization

The phase structures of the samples were investigated via X-ray diffraction. The grain morphology was investigated by SEM. Finer microscopic morphology was investigated by TEM, with energy dispersive spectroscopy (EDS) to analyze the elemental composition and distributions of the samples. The elemental composition was analyzed using EPMA.

### Thermoelectric property measurements

The electrical and thermal transport properties were all measured in the same direction as perpendicular to the uniaxial SPS pressure. The *σ* and *S* measurement were conducted using a ZEM-3 apparatus (Ulvac-Riko, Japan). The thermal diffusion coefficient (*D*) is measured by the laser flash method (Netzsch LFA 457, Germany). *κ* can be further obtained via the equation *κ* =* DC*_p_*d*, where *C*_p_ is the specific heat deduced via the Dulong–Petit limit and *d* is the density measured by the Archimedes method. The electrical thermal conductivity (*κ*_e_) was calculated via the Wiedeman–Franz law *κ*_e_ =* σLT*. The Hall coefficient (*R*_H_) measurement via the van der Pauw method. According to the equation *n*_H_ = 1*/(eR*_H_) and *μ*_H_ =* σR*_H_, *n*_H_ and *μ*_H_ were obtained. The single-leg modules for *η* were measured via a Mini-PEM testing system (Ulvac-Riko, Japan). The theoretical conversion efficiency was simulated via COMSOL Multiphysics software. In comparison with TE power generation, there are no standardized methods for measuring cooling performance [53]. Therefore, Δ*T*_max_ of the micro PCs were visually assessed using commercial equipment called Z-Meters (RMT Ltd., Russia) [54], which follows the Harman approach [55]. Since Z-Meters have been extensively used to measure the Δ*T*_max_ [36,38,39], our measurement results are valid for comparison with previously reported data. In addition, the COP was measured using homemade equipment set up by Jianju Technology Co., Ltd.

### Mechanical property measurements

All specimens for the flexural and compressive test were cut along the direction perpendicular to the uniaxial SPS pressure with careful polishing. The test was conducted on an MTS universal test machine (E44.104, MTS, China).

## Supplementary Material

nwae329_Supplemental_File
