# Supplementary material for: Strong and efficient bismuth telluride-based thermoelectrics for Peltier microcoolers
Source: Natl Sci Rev. 2024 Sep 16;11(10):nwae329. doi: 10.1093/nsr/nwae329 (PMC11495490; doi:10.1093/nsr/nwae329)
Supplement: nwae329_Supplemental_File [file nwae329_supplemental_file.pdf]

Supporting Information

Strong and efficient bismuth telluride-based thermoelectrics for Peltier microcoolers

Hua-Lu Zhuang[1], Bowen Cai[2], Yu Pan[3], Bin Su[1], Yilin Jiang[1], Jun Pei[1], Fengming Liu[2], Haihua Hu[1], Jincheng Yu[1], Jing-Wei Li[1], Zhengqin Wang[1], Zhanran Han[1], Hezhang Li[1,4], Chao Wang[4] and Jing-Feng Li[1,*]

[1]State Key Laboratory of New Ceramics and Fine Processing, School of Materials Science and Engineering, Tsinghua University, Beijing 100084, China;

[2]Guangxi Pilot Free Trade Zone Jianju Technology Co., LTD., Qinzhou 535000, China;

[3]Max Planck Institute for Chemical Physics of Solids, Dresden 01187, Germany;

[4]Department of Precision Instrument, Tsinghua University, Beijing 100084, China.

Corresponding author. E-mail: jingfeng@mail.tsinghua.edu.cn

1. Detailed materials and methods

Synthesis

Bi (powder, 99.99%), Sb (granules, 99.99%) and Te (powder, 99.999%), SiC (powder, 99.99%, 30 nm) were used as the starting materials, which were weighed and loaded into stainless steel jars in a glove box according to the stoichiometric ratio of $Bi_{0.4}Sb_{1.6}Te_3$, $Bi_{0.4}Sb_{1.6}Te_3$ - 0.4 vol% SiC and $Bi_{0.4}Sb_{1.6}Te_{3.01}$ - 0.4 vol% SiC. The SiC content of 0.4 vol% was determined according to the optimal volume fraction reported in our previous study [1,2]. The XRD pattern and TEM image of the SiC nanoparticles are shown in **Fig. S26**. The starting materials were first mixed and initially reacted via the mechanical alloying (MA) process by setting the jars on a planetary ball-milling machine for 360 min at 480 rpm with a high-purity argon atmosphere inside. The MAed powders were then poured into graphite dies of 12 mm diameter and sintered by spark plasma sintering (SPS) at 673 K under a pressure of 50 MPa in a vacuum (< 5 Pa) for 5 min. The MA-SPSed bulks were sealed into quartz tubes with $< 5\times10^{-3}$ Pa inside. The annealing process was operated in a muffle oven. Finally, the annealed bulks were loaded into a graphite die of a larger size of 15 mm diameter and hot-forged (HF) by SPS at 673 K under a pressure of 50 MPa in a vacuum (< 5 Pa) for 15 min. The final $Bi_{0.4}Sb_{1.6}Te_3$ sample annealed at 673 K for 6 h, 12 h, 24 h and 48 h were labeled as 673A6, 673A12, 673A24 and 673A48, respectively. The $Bi_{0.4}Sb_{1.6}Te_3$ incorporated with 0.4 vol% nano SiC with different annealing durations were labeled as 673A12S, 673A24S and 673A48S, respectively. After adding excess Te, the $Bi_{0.4}Sb_{1.6}Te_{3.01}$ incorporated with 0.4 vol% nano SiC with different annealing temperatures were labeled as 673A48ST, 723A48ST, 773A48ST, 823A48ST and 873A48ST, respectively. The process of SPS, annealing and hot-forging (A-HF) are schematically shown in **Fig. S1**. The bulk subjected to HF under the same condition but without annealing was taken as the control group, labeled as A0.

The micro cuboid pillar arrays were processed using a dicing saw on slices of about $1.5 \times 1.5 \times 0.5$ mm^3. The blade thickness was 100 μm, and it rotated at a speed of 30,000 rpm. The cooling water flow rate is set at 0.3 L/min. The step distances for each cut were 0.15 and 0.17mm, respectively. The feeding speed was 0.1 mm/s. Consequently, the total time required for processing a single sample amounted to approximately 1 h.

The micro Peltier coolers (PCs) were fabricated by Jianju Technology Co., Ltd. The sample 823A48ST was selected to fabricate the p-leg of the micro PCs, i.e., the Sample 4 in the manuscript. The n-leg of the micro PCs was prepared from the n-type Bi_2Te_3 sourced from Jianju Technology Co., Ltd. Some specific information about the n-type Bi_2Te_3 can be found in Section 8 of the Supporting Information. Initially, the bulk samples were sliced into 0.4 mm thickness plates. After polishing the slice surfaces, a Ni coating with a thickness of ~3 μm was applied through electroplating. Subsequently, the slices were further cut using a dicing saw to obtain cubic pillars, which were then picked up by an automated manipulator and placed on an AlN ceramic plate attached with Cu electrodes coated with Sn-Ag-Cu solder paste. The thermoelectric (TE) leg was then welded to the Cu electrode through reflow welding. The photograph and elemental distribution of the interface between Sample 4, the diffusion barrier layer and the solder are provided in **Fig. S28**, demonstrating good wetting and bonding between these layers. Next, another AlN ceramic plate attached with Cu electrodes coated in welding paste was placed on the other end of the TE legs, and the complete micro PCs were obtained through reflow welding again.

Structural characterization

The phase structures of the samples were investigated via X-ray diffraction (XRD, Cu Kα, Rigaku 2500, Japan). The grain morphology was investigated by a field-emission scanning electron microscope (FESEM, Zeiss Merlin, Germany). Finer microscopic morphology was investigated by transmission electron microscope (TEM, 2100F, JEOL, Japan), with an energy-dispersive X-ray spectrometer (EDS) to analyze the elemental composition and distributions of the samples. The elemental composition was analyzed using an electronic probe microanalyzer (EPMA; JXA-8230, JEOL, Japan).

Thermoelectric property measurements

The electrical and thermal transport properties were all measured in the same direction as perpendicular to the uniaxial SPS pressure. The HFed bulks were cut and polished into different shapes and sizes for various measurements. The bars of 2 mm × 2 mm × 10 mm were used for electrical conductivity (σ) and Seebeck coefficient (S) measurement via a ZEM-3 apparatus (Ulvac-Riko, Japan) using a four-probe method in a helium atmosphere. Temperature gradients of 10 K, 20 K, and 30 K were applied for the ZEM-3 measurement. The discs of φ 6 mm × 1 mm were used for thermal diffusion coefficient (D) measurement by the laser flash method (Netzsch LFA 457,

Germany). The total thermal conductivity (κ) can be further obtained via the equation $\kappa = DC_pd$, where C_p is the specific heat deduced via the Dulong-Petit limit and d is the density measured by the Archimedes method. The electrical thermal conductivity (κ_e) was calculated via the Wiedeman-Franz law $\kappa_e = \sigma LT$, where the Lorenz factor (L) was roughly calculated by the equation $L = 1.5 + \exp(-\frac{|S|}{116})$ [3]. The pieces of 5 mm × 5 mm × 1 mm were used for Hall coefficient (R_H) measurement by a Hall measurement system (ResiTest 8340DC, Toyo, Japan) via the van der Pauw method. According to the equation $n_H = 1/(eR_H)$ and $\mu_H = \sigma R_H$, the Hall carrier concentration (n_H) and mobility (μ_H) were obtained. The bulks of 5 mm × 5 mm × 4 mm were used for a simple assembly without complicated welding of the single-leg modules for conversion efficiency (η) measurement via the Mini-PEM testing system (Ulvac-Riko, Japan), the details of which were shown in our previous work [4]. The theoretical conversion efficiency was simulated via the COMSOL Multiphysics software. In comparison with TE power generation, there are no standardized methods for measuring cooling performance [5]. Therefore, maximum cooling temperature difference values (ΔT_{max}) of the micro PCs were visually assessed using a commercial equipment called Z-Meters (RMT Ltd., Russia) [6], which follows the Harman approach [7]. Since Z-Meters has been extensively used to measure the ΔT_{max} [8–10], our measurement results are valid for comparison with previously reported data. In addition, the cooling coefficient of performance (COP) was measured using a homemade equipment set up by Jianju Technology Co., Ltd.

Mechanical property measurements

All specimens for the flexural and compressive test were also cut along the direction perpendicular to the uniaxial SPS pressure with careful polishing. The test was conducted on an MTS universal test machine (E44.104, MTS, China). The bars of 2 mm × 2 mm × 15 mm were used for the 3-point bending test to obtain flexural strength. The bulks of 3 mm × 3 mm × 6 mm were used for the compression test.

2. TE performance of samples fabricated using MA-SPS and A-HF with different annealing durations

The A-HF parameter and sample composition were systematically investigated with several samples. All samples exhibited the well-indexed main phase of the pure $(Bi,Sb)_2Te_3$ phase (see the XRD pattern of samples in **Fig. S7**). The photos of the swelled samples after annealing for different parameters are demonstrated in **Fig. S8**. The density of the final obtained samples is demonstrated in **Table S1**.

Firstly, to investigate the annealing duration for the A-HF process, the annealing temperature of 673 K was selected for the MA-SPSed $Bi_{0.4}Sb_{1.6}Te_3$ sample. The temperature dependence of the electrical transport properties for the samples subjected to different annealing durations is exhibited in **Fig. S9**. All samples show typical characteristics of degenerate semiconductors that the σ monotonically decreases with increasing temperature. Generally, the annealed samples always possess higher σ than those without annealing. Nevertheless, the σ does not monotonically increase with the increasing annealing duration. The 673A12 sample shows the highest σ among the 673 K annealed samples. When the annealing duration further increases, the σ gradually decreases. **Fig. S9b** shows that the annealed sample exhibits a lower S than the sample without annealing in general, while the S does not show obvious dependence on the annealing duration. Reversely, the S seems sensitive to the annealing temperature that higher annealing temperature would lead to lower S. In addition, the S of all samples shows the same trend increasing firstly and then decreasing owing to the intrinsic excitation of narrow bandgap semiconductors. Comprehensively, the synchronous change of σ and S leads to an improved power factor (PF) for the annealed sample, as shown in **Fig. S9c**. When the annealing duration is less than 12 h, the increase of annealing duration is beneficial to PF enhancement, while further prolonging the annealing duration deteriorates the PF.

The variation of electrical transport properties can be further clarified by the n_H and μ_H, as shown in **Fig. S9d and e**. The n_H generally increases with the increasing annealing duration and temperature, which contributed a lot to the increased σ. The increase in n_H can be attributed to the increasing number of antisite defects (Sb'_{Te} and Bi'_{Te}) during annealing, which have lower formation energy compared to other defects [11]. The increased n_H also contributes to the decline in S. However, the increased antisite defect can also enhance carrier scattering. Therefore, there must be another

reason for the simultaneous increase in μ_{H}. Although there are no significant changes to the average grain size (**Fig. S10**), it is speculated that some nanograins may merge into larger grains during annealing, thereby weakening the grain boundary scattering. In addition, the μ_{H} first increases and then decreases with the increasing annealing duration, being maximized in the 673A12 sample. Different from σ, the increase of charge carrier concentration and mobility impair the S. For degenerate semiconductors, S can be expressed through the Mott equation [12]:

$$S = \frac{\pi^2 k_{\mathrm{B}}^2 T}{3e}\left\{\frac{1}{n}\frac{dn(E)}{dE} + \frac{1}{\mu}\frac{d\mu(E)}{dE}\right\}_{E=E_{\mathrm{F}}} \tag{1}$$

where k_{B}, e and E_{F} are the Boltzmann constant, elementary charge and Fermi energy, respectively. The magnitude of S still exhibits a negative correlation with charge carrier concentration. Comparing the variation of n_{H} and S, the change of S with the annealing condition is not as distinct as the trends of n_{H}, which may be attributed to the competition of the charge carrier concentration and mobility. Therefore, it is assumed that the samples mentioned in this work can be analyzed by the single parabolic band (SPB) model [13,14]. Furthermore, according to a Drude-Sommerfeld free electron model deduced by Snyder *et al.*, the weighted mobility (μ_{W}) can be easily calculated by measured σ and S, which reflects the more intrinsic ability for charge carrier transport excluding the effect of density of electron states [15]:

$$\mu_{\mathrm{W}} = \frac{3h^3\sigma}{8\pi e(2m_e k_{\mathrm{B}}T)^{\frac{3}{2}}}\left[\frac{\exp\left[\frac{|S|}{k_{\mathrm{B}}/e}-2\right]}{1+\exp\left[-5\left(\frac{|S|}{k_{\mathrm{B}}/e}-1\right)\right]} + \frac{\frac{3}{\pi^2}\frac{|S|}{k_{\mathrm{B}}/e}}{1+\exp\left[5\left(\frac{|S|}{k_{\mathrm{B}}/e}-1\right)\right]}\right] \tag{2}$$

where h and m_{e} are the Plank constant and electronic mass, respectively. As shown in **Fig. S9f**, the μ_{W} exhibits a similar variation as μ_{H} with the different annealing conditions, meaning that the charge carrier transport is actually improved after adding the annealing process before the HF process.

For the $(Bi,Sb)_2Te_3$ subjected to HF and long-time annealing, the crystalline orientation and grain size may be other contributing factors to the improved charge carrier transport. However, these two factors can be easily ruled out. **Fig. S11a** presents the XRD patterns along the direction parallel to the SPS pressure for samples subjected to the A-HF process with different annealing durations, which are well indexed to the pure phase of $(Bi,Sb)_2Te_3$ (PDF #49-1713). The orientation factor (F) is calculated by the Lotgering method [16]:

$$F = \frac{P - P_0}{1 - P_0} \tag{3}$$

$$P_0 = \frac{\sum I_0(00l)}{\sum I_0(hkl)} \tag{4}$$

$$P = \frac{\sum I(00l)}{\sum I(hkl)} \tag{5}$$

where I and I_0 are the intensities of the corresponding crystallographic planes for the preferentially (for (00l) plane) and randomly oriented samples, respectively. As shown in **Fig. S11b**, it is independent of the crystalline orientation and annealing condition.

As shown in **Fig. S12a**, the variation trend of κ with temperature for all samples is similar, indicating that the bipolar effect is not obviously changed after the A-HF treatment. The κ_L for the samples annealed before HF is generally lower than the sample subjected to the HF process alone. In general, the κ_L is reduced after the A-HF process. The 673A48 sample shows the lowest κ_L. Therefore, it can be concluded that the annealing process is beneficial to the reduction of κ_L. To understand the intrinsic reason for the reduction, transmission electron microscope (TEM) analysis has been conducted on the 673A48 sample (**Fig. S13**). As observed from the TEM, the reason may be the generated nano distortions and dislocations after the A-HF process. These microstructures are related to the enhancement of phonon scattering, thereby beneficial to the reduction of κ_L. As demonstrated in **Fig. S12b**, all samples subjected to A-HF process show improved ZT values. The 673A48 sample shows the highest ZT value in the whole temperature range, of which the maximum ZT value is 1.34.

3. TE performance of SiC-incorporated samples fabricated using MA-SPS and A-HF with different annealing durations

Considering the independent nature of the A-HF process as a posttreatment method, it has the potential to synergize with pretreatment processes such as doping and nano-composition. In our previous study, the strategy of nano SiC composite can simultaneously improve the TE and mechanical properties of $(Bi,Sb)_2Te_3$. The additive of 0.4 vol% is always the best [1,2]. Therefore, in this work, we further incorporated 0.4 vol% nano SiC as a raw material. The MA-SPSed bulk was respectively annealed at 673 K for 0, 24 and 48 h, and then undergoing HF treatment. The TE performance of these samples is presented in **Fig. S14**, which demonstrates a similar trend to pure $Bi_{0.4}Sb_{1.6}Te_3$. Notably, the 673A48S sample exhibits superior TE performance with a maximum *ZT* value reaching up to 1.42 and an average *ZT* value (300 - 500 K) achieving 1.26.

4. TE performance of the SiC-incorporated and Te-excess samples fabricated using MA-SPS and A-HF with different annealing temperatures

The temperature difference of electrical transport properties for the A-HF sample with different annealing temperatures is demonstrated in **Fig. S15**. The observed variation trend with test temperature remains consistent across different annealing temperatures, suggesting that the band structure and band gap may not undergo significant changes. In general, σ increases with increasing annealing temperature, while S decreases to varying degrees. Compared to the 673A48ST sample, samples subjected to higher annealing temperatures exhibit higher PF values. Notably, the 823A48ST sample demonstrates the highest PF value. The Hall measurement results partially explain these variations: n_H generally increases with increasing annealing temperature, whereas μ_H does not show obvious changes. However, the μ_H does not obviously change with increasing annealing temperature. These factors offset the expected reduction in carrier mobility due to increased carrier concentration. As shown in **Fig. S16**, it is possible that an increase in grain size resulting from higher annealing temperatures facilitates charge carrier transport and contributes to an increase in σ. Despite a slight decrease in S attributed to improved microstructure for charge carrier transport, its impact on PF is negligible. Overall, optimization of defects and microstructure through increased annealing temperature enhances electrical transport properties.

The temperature difference in thermal transport properties for the A-HF sample with different annealing temperatures is demonstrated in **Fig. S17a and b**. Attributed to the increased n_H, the κ slightly increased with the increase of annealing temperature. However, samples with higher annealing temperatures exhibit slightly lower κ_L. This can be attributed to the increased dislocation density observed in the TEM images shown in **Fig. 4c and Fig. S24**, which enhances the scattering of mid-frequency phonons. The higher dislocation density may result from larger volume deformation, which is attributed to more significant expansion caused by higher annealing temperatures. Although there is a slight deterioration in total thermal transport properties, the improvement of electrical transport properties contributes to an enhanced *ZT* value (**Fig. S17c**). The maximum achieved *ZT* value was 1.50 at 348 K.

5. TE performance of the samples without annealing

To avoid the potential influence of the HF and elongated heat preservation process on the analysis of TE performance, the TE performance for the sample sintered at 673 K for 5 min, the sample sintered at 673 K for 20 min, and the sample sintered at 673 K for 5 min and then followed by HF for 15 min are compared in **Fig. S18**. Remarkably, all these samples exhibited similar TE performance, suggesting that both the HF process and slight extension in sintering time have negligible effects on the TE performance of $Bi_{0.4}Sb_{1.6}Te_3$.

6. Calculation of the electronic quality factor

The electronic quality factor (B_E) is calculated from the measured S and σ from the following equation[17]:

$$B_E = S^2\sigma/[\frac{S_r^2 \exp(2-S_r)}{1+\exp[-5(S_r-1)]} + \frac{S_r\pi^2/3}{1+\exp[5(S_r-1)]}] \quad (6)$$

where $S_r = \frac{|S|}{k_B/e}$.

7. Discussion of the measured and simulated results of the single-leg device

The measured and simulated results of the single-leg device are shown in **Figs S19 and S20**, respectively. In general, the variation trend of measured and simulated results is the same; however, as shown in **Fig. S20a**, the simulated η is higher than the measured η, especially under a high temperature difference (ΔT).

The output voltage-current (*V-I*) curves exhibit a linear relationship, while the output power-current (*P-I*) curves exhibit a parabolic relationship. The slope and intercept of the *V–I* curve represent the internal resistance and open-circuit voltage of the single-leg device, respectively. The open-circuit voltage, output power and heat flow increase with increasing ΔT. The measured open-circuit voltage rises from 5.7×10^{-3} to 4.5×10^{-2} V, and the measured internal resistance increases from 5.0×10^{-3} to 5.3×10^{-3} Ω (**Fig. S19a**). In contrast, the simulated open-circuit voltage rises from 6.0×10^{-3} to 5.1×10^{-2} V, and the measured internal resistance increases from 3.2×10^{-3} to 5.5×10^{-3} Ω (**Fig. S20b**). Overall, these findings indicate lower open-circuit voltage and larger internal resistance in our experimental measurements compared to simulations, which are attributed to the interface thermal resistance and interface resistance introduced by the contact and soldering between the sample, diffusion barrier layers and electrodes, leaving room for further optimization.

At ΔT = 225 K, the measured and simulated output power are 9.6×10^{-2} W and 11.7×10^{-2} W, respectively (**Figs S19b and S20c**). The discrepancy between the measured and simulated output power can be attributed to the lower actual temperature difference stemming from interface thermal resistance, as well as higher internal resistance affecting the external work of the device. This higher internal resistance is also reflected in a lower current corresponding to peak output power.

In addition, the measured heat flow at each ΔT showed larger values compared to simulated results, which has negative impacts on η (**Figs S19c and S20d**); this may be due to heat radiation between hot and cold sides during measurement.

Therefore, the optimization of fabrication processes and device structures should focus on improving diffusion barrier layers and soldering processes, so as to reduce interface thermal resistance and interface resistance while securing low heat flow between hot and cold sides.

8. Information on the n-type Bi_2Te_3 used in micro PCs

The n-type Bi_2Te_3 used in micro PCs was provided by Jianju Technology Co., Ltd. The n-type Bi_2Te_3 was prepared using a hot extrusion method independently designed by Jianju Technology Co., Ltd. As shown in **Fig. S29**, XRD analysis revealed diffraction peaks corresponding to the rhombohedral phase of Bi_2Te_3 (PDF #15-0863). Based on the XRD patterns measured perpendicular to the extrusion direction, the orientation factor can be calculated using equations (3) to (5), demonstrating the development of slight texture with an orientation factor of approximately 0.12. This texture is also distinguishable on the fracture surface as shown in SEM image in **Fig. S30**. Additionally, the average grain size of the n-type Bi_2Te_3 was determined to be approximately 8 μm. Through EDS component analysis, it was found that the atomic ratio of Bi:Te in the actual component is about 43.71:56.29. The TE performance of the n-type Bi_2Te_3 is presented in **Fig. S4**, where σ exhibits a monotonically decreasing trend with increasing temperature, indicating typical characteristics of a non-degenerate semiconductor material. After reaching 323 K, both Seebeck coefficient and thermal conductivity show an inflection point suggesting the possible occurrence of bipolar diffusion effect which adversely affects TE properties. Consequently, the peak *ZT* value of 1.24 is observed at 323 K.

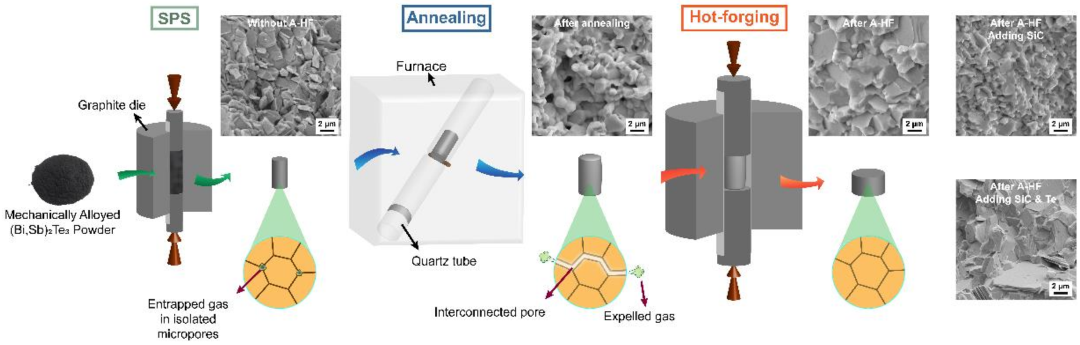

Figure S1. The schematic diagram showing the annealing process followed by HF.

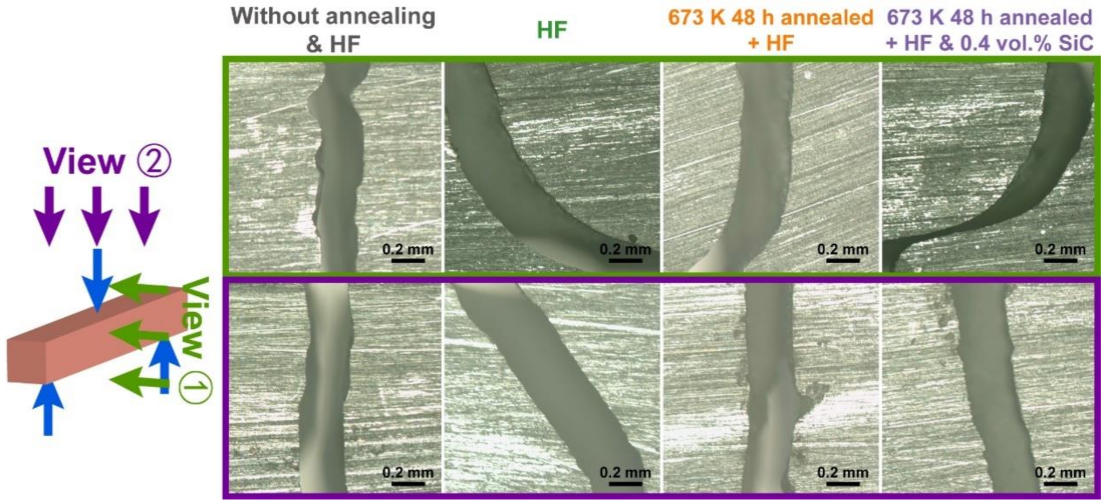

Figure S2. Optical microscopy images of some representative specimens used for flexural strength measurement. Fracture appearance of $Bi_{0.4}Sb_{1.6}Te_3$ in this work at different stages.

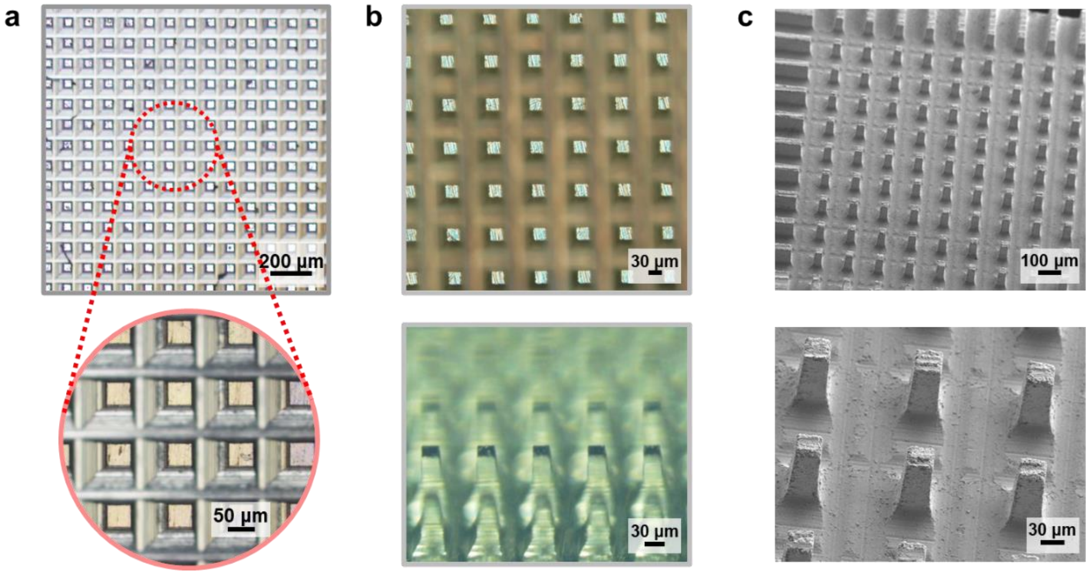

Figure S3. Microscopy images of the diced surface. The produced micro cuboid pillar arrays on the surface of **(a)**,**(b)** the 673A48S and **(c)** the improved 823A48ST sample using a dicing saw.

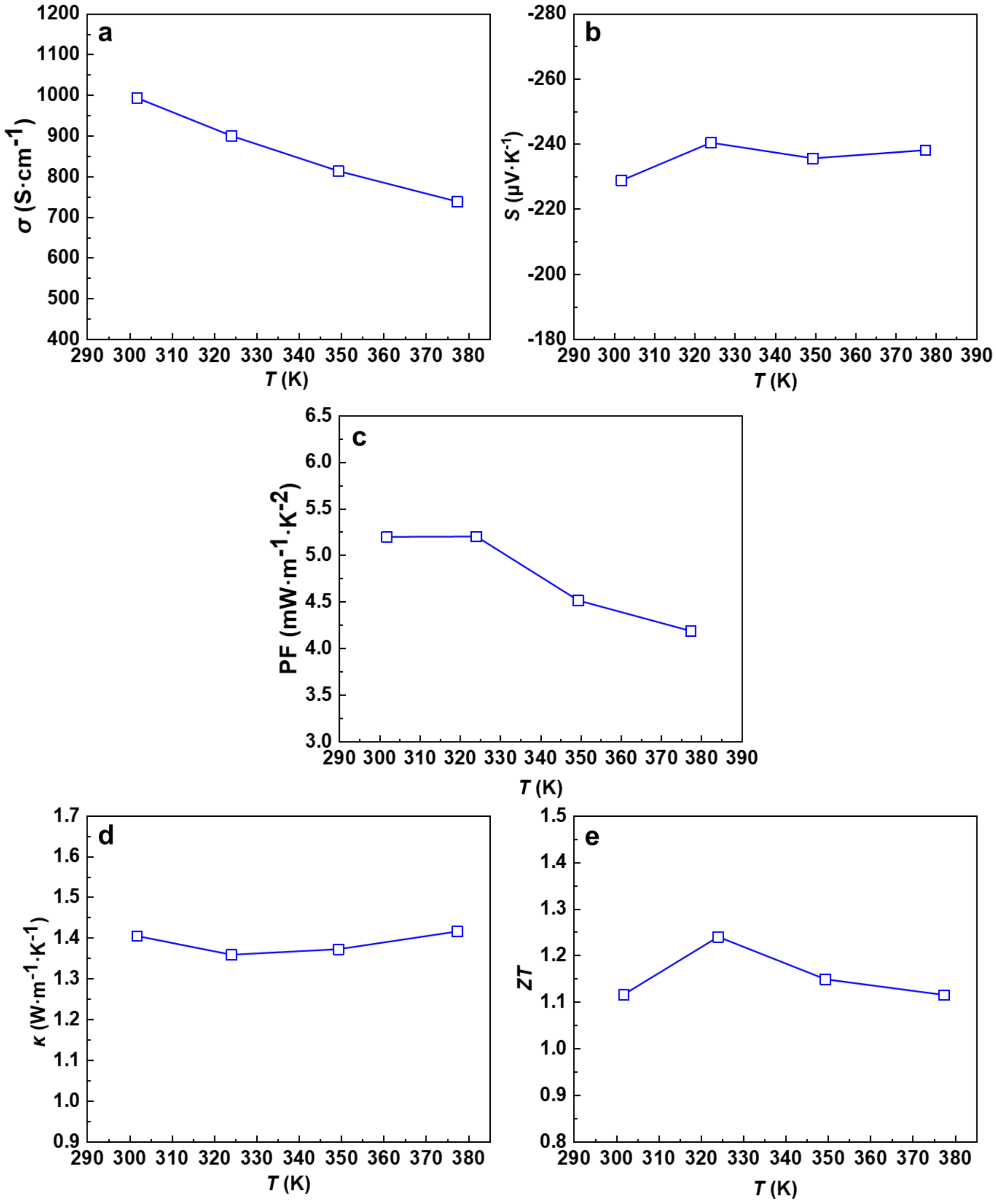

Figure S4. TE performance of the n-type Bi_2Te_3. Temperature dependence of **(a)** electrical conductivity, **(b)** Seebeck coefficient, **(c)** power factor, **(d)** total thermal conductivity and **(e)** *ZT* value for the n-type Bi_2Te_3 materials used for micro PCs.

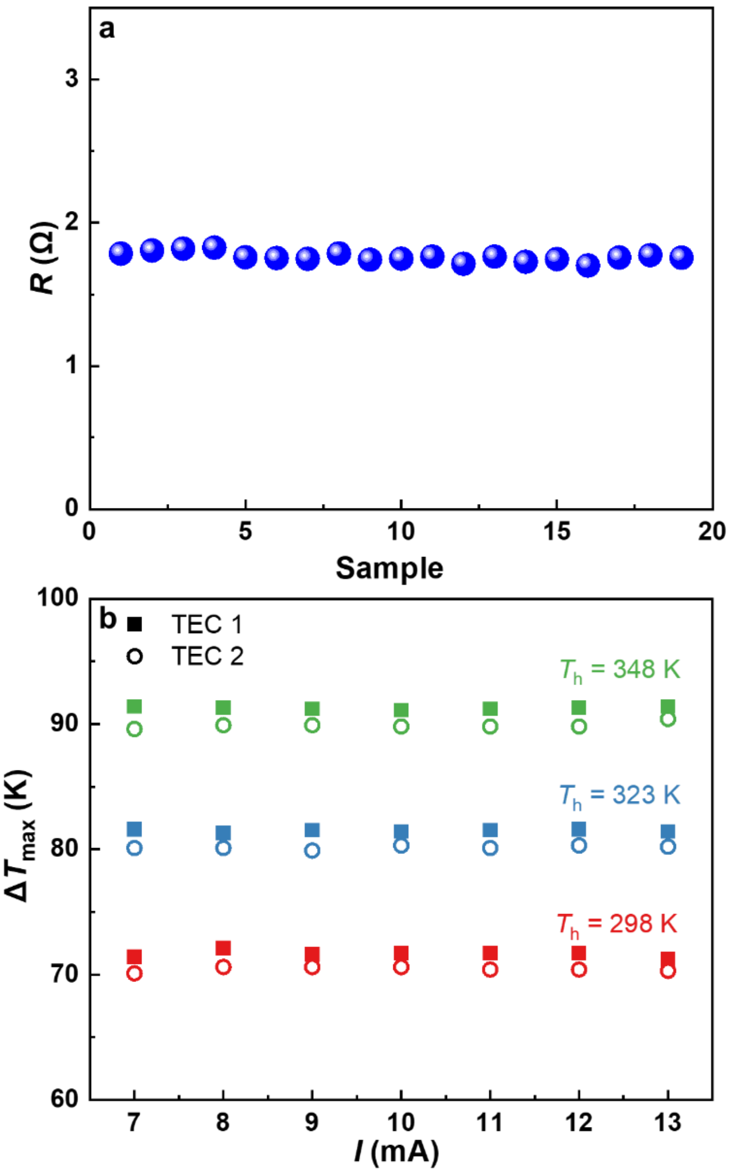

Figure S5. The reliability of the fabrication process and the micro PCs. (a) The measured AC internal resistance for 19 prepared micro PCs. **(b)** The maximum cooling temperature of two micro PCs as a function of test current at T_h = 298 K, 323 K and 348 K.

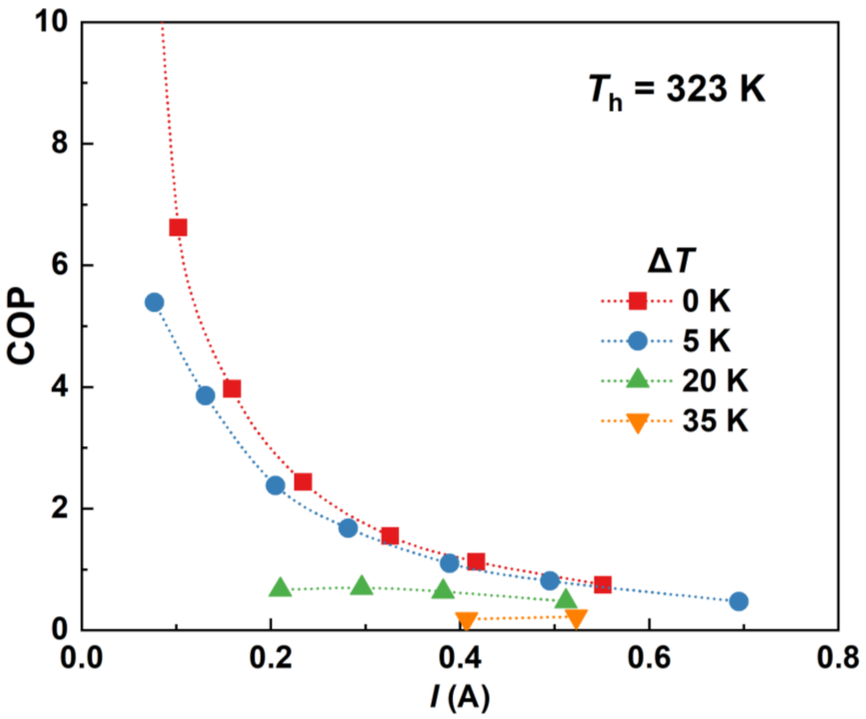

Figure S6. The COP of the micro PCs. The measured COP as a function of electric current I at the ΔT of 0, 5, 20, and 35 K when T_h is 323 K.

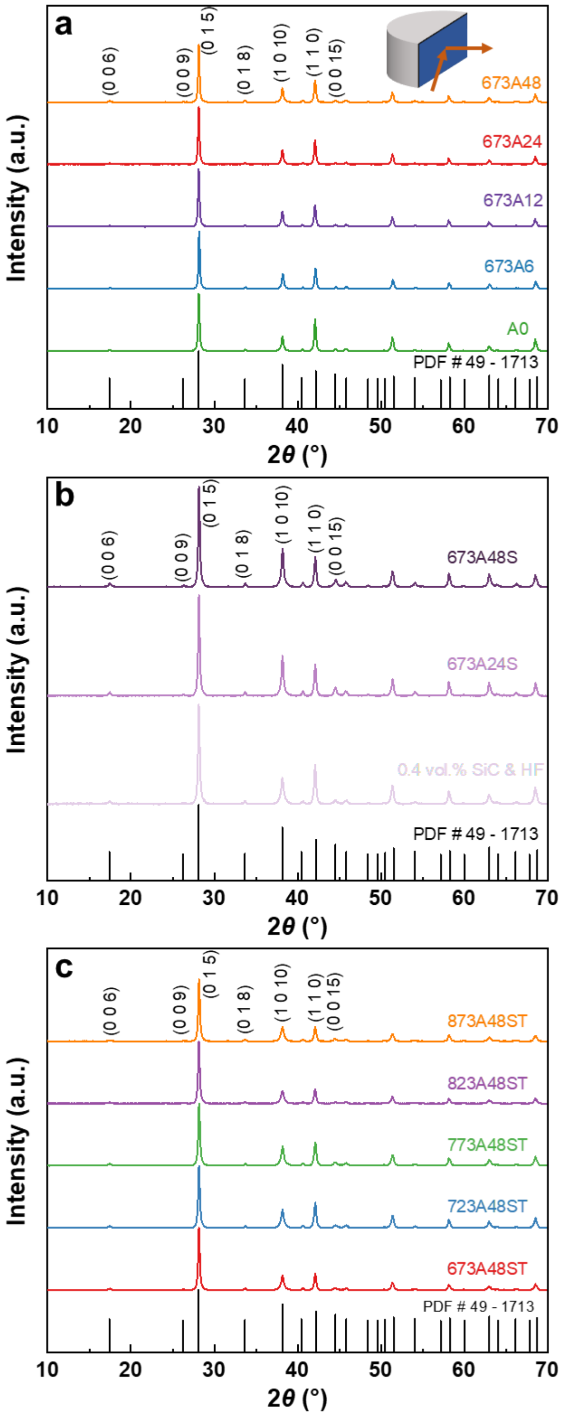

Figure S7. XRD patterns of the (Bi,Sb)$_2$Te$_3$-based samples. XRD patterns of **(a)** the $Bi_{0.4}Sb_{1.6}Te_3$ samples and **(b)** the 0.4 vol% SiC-incorporated $Bi_{0.4}Sb_{1.6}Te_3$ samples subjected to annealing with different durations at 673 K followed by HF, as well as the **(c)** 0.4 vol% SiC-incorporated $Bi_{0.4}Sb_{1.6}Te_{3.01}$ samples subjected to annealing at different temperatures for 48 h and followed by HF.

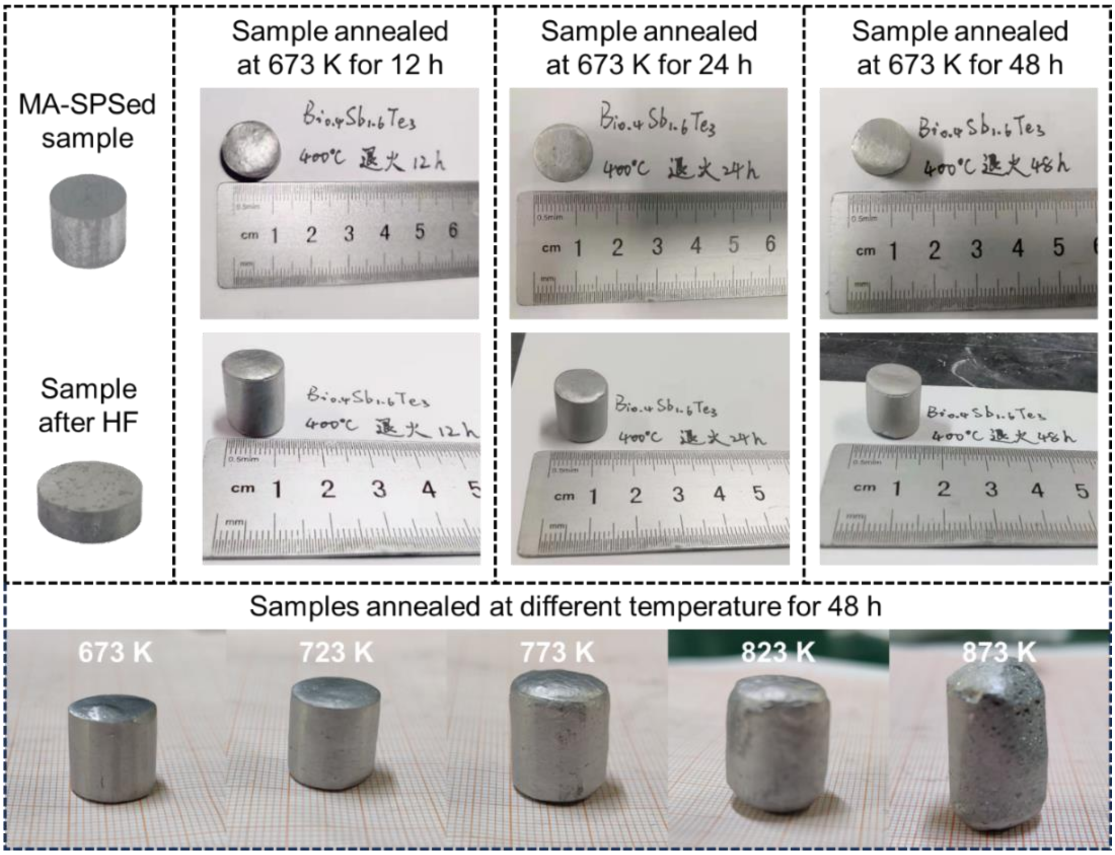

Figure S8. The photos taken from the samples prepared under different conditions.

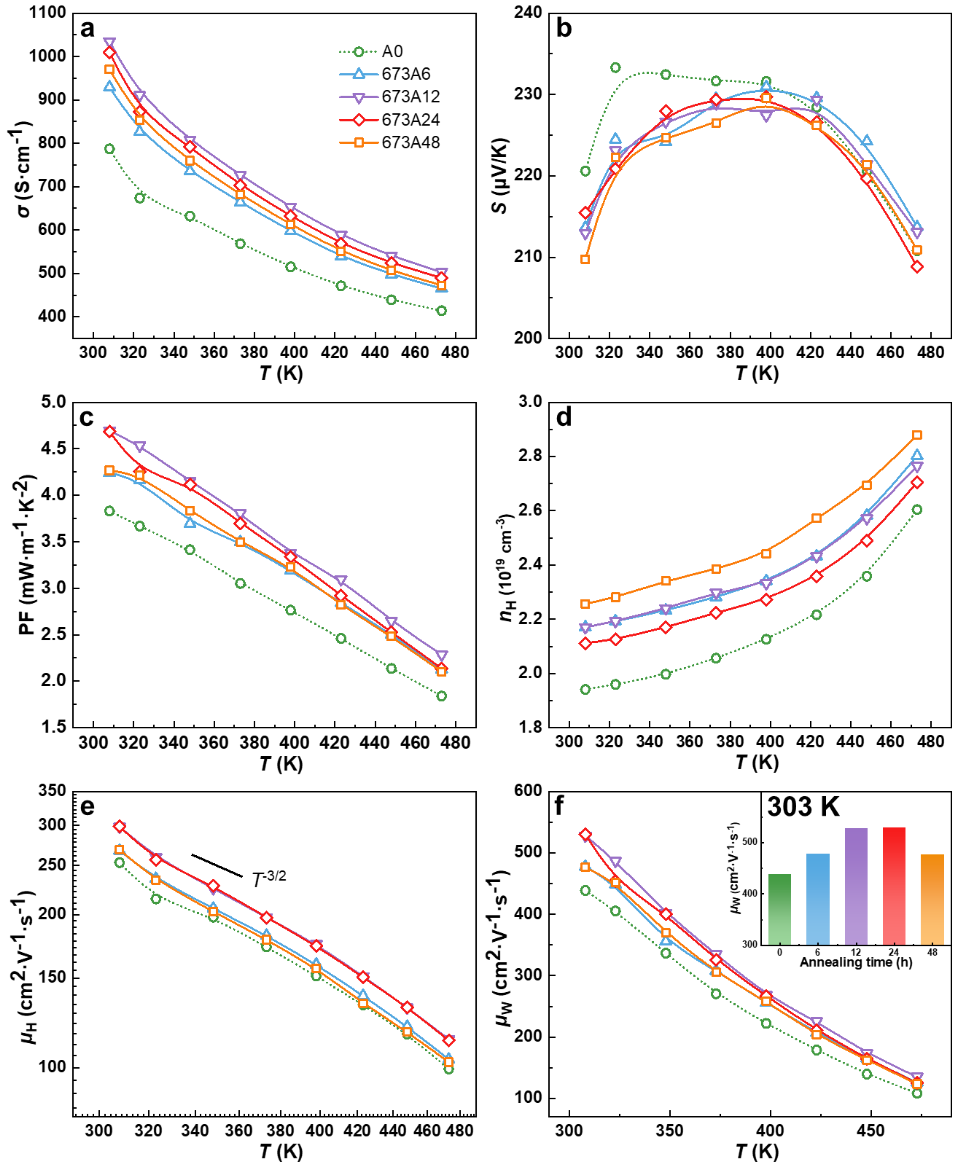

Figure S9. Electrical transport properties of the $Bi_{0.4}Sb_{1.6}Te_3$ samples with different annealing durations. Temperature dependence of **(a)** electrical conductivity, **(b)** Seebeck coefficient, **(c)** power factor, **(d)** Hall carrier concentration, **(e)** Hall mobility and **(f)** weighted mobility (inset shows the weighted mobility values at 303 K) for $Bi_{0.4}Sb_{1.6}Te_3$ samples subjected to annealing with different durations at 673 K followed by HF.

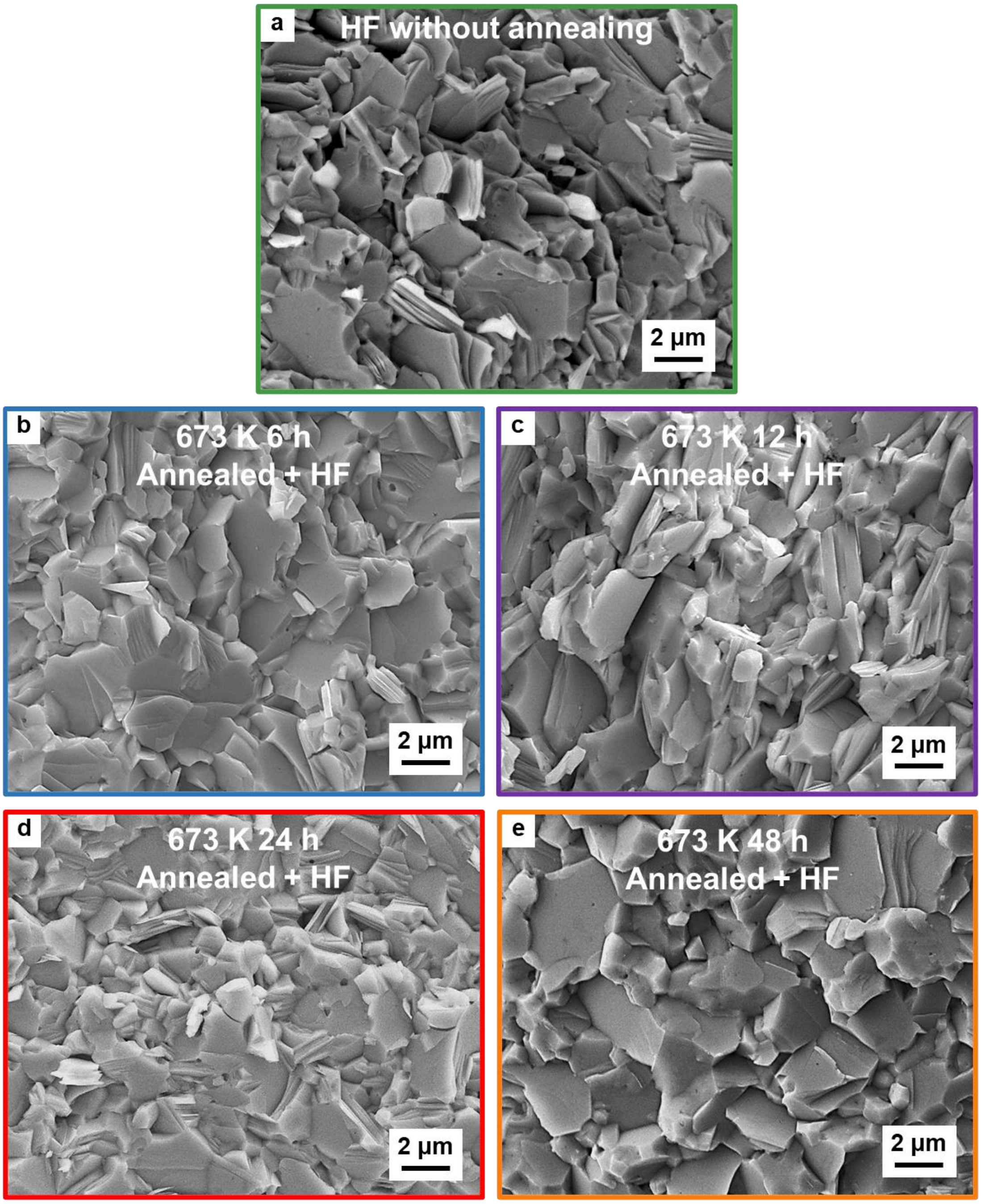

Figure S10. SEM images of the $Bi_{0.4}Sb_{1.6}Te_3$ samples with different annealing durations. (a)-(e) The SEM images of the fracture surface for the $Bi_{0.4}Sb_{1.6}Te_3$ samples subjected to annealing with different durations at 673 K followed by HF.

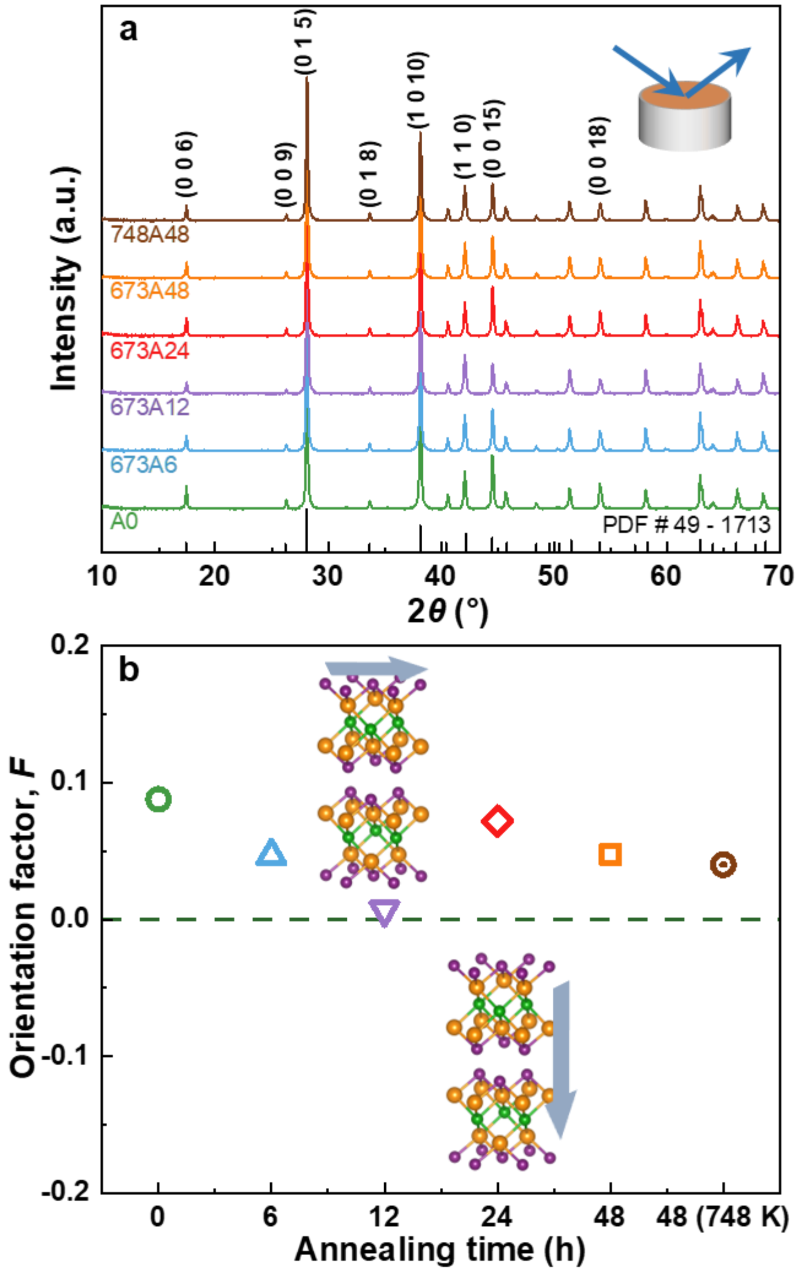

Figure S11. XRD patterns and orientation factors of the $Bi_{0.4}Sb_{1.6}Te_3$ samples with different annealing durations. (a) The XRD patterns for $Bi_{0.4}Sb_{1.6}Te_3$ samples subjected to annealing with different durations at 673 K followed by HF along the direction parallel to the direction of SPS pressure. **(b)** The orientation factors of the (00*l*) plane calculated from the XRD patterns shown in **(a)**.

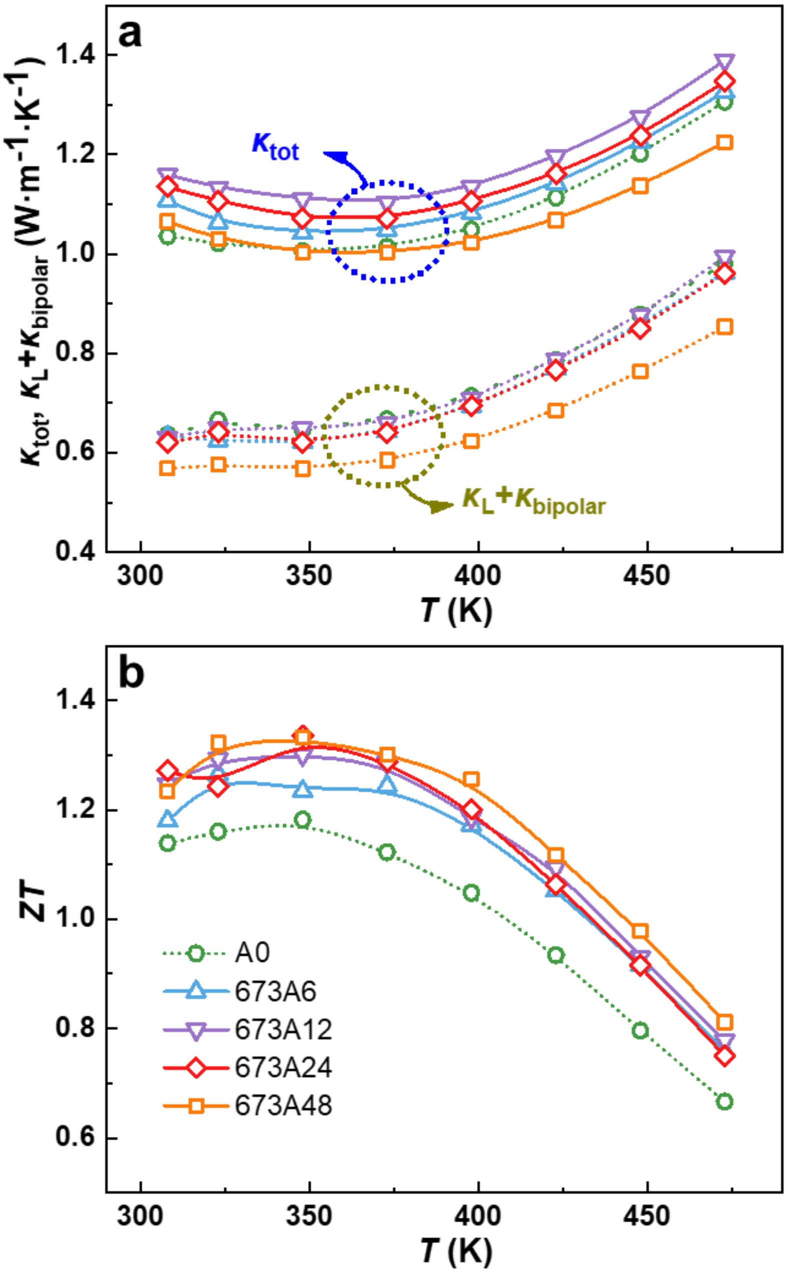

Figure S12. Thermal transport properties and *ZT* values of the $Bi_{0.4}Sb_{1.6}Te_3$ samples with different annealing durations. Temperature dependence of **(a)** total thermal conductivity and lattice plus bipolar thermal conductivity, as well as **(b)** *ZT* values for $Bi_{0.4}Sb_{1.6}Te_3$ samples subjected to annealing with different durations at 673 K followed by HF.

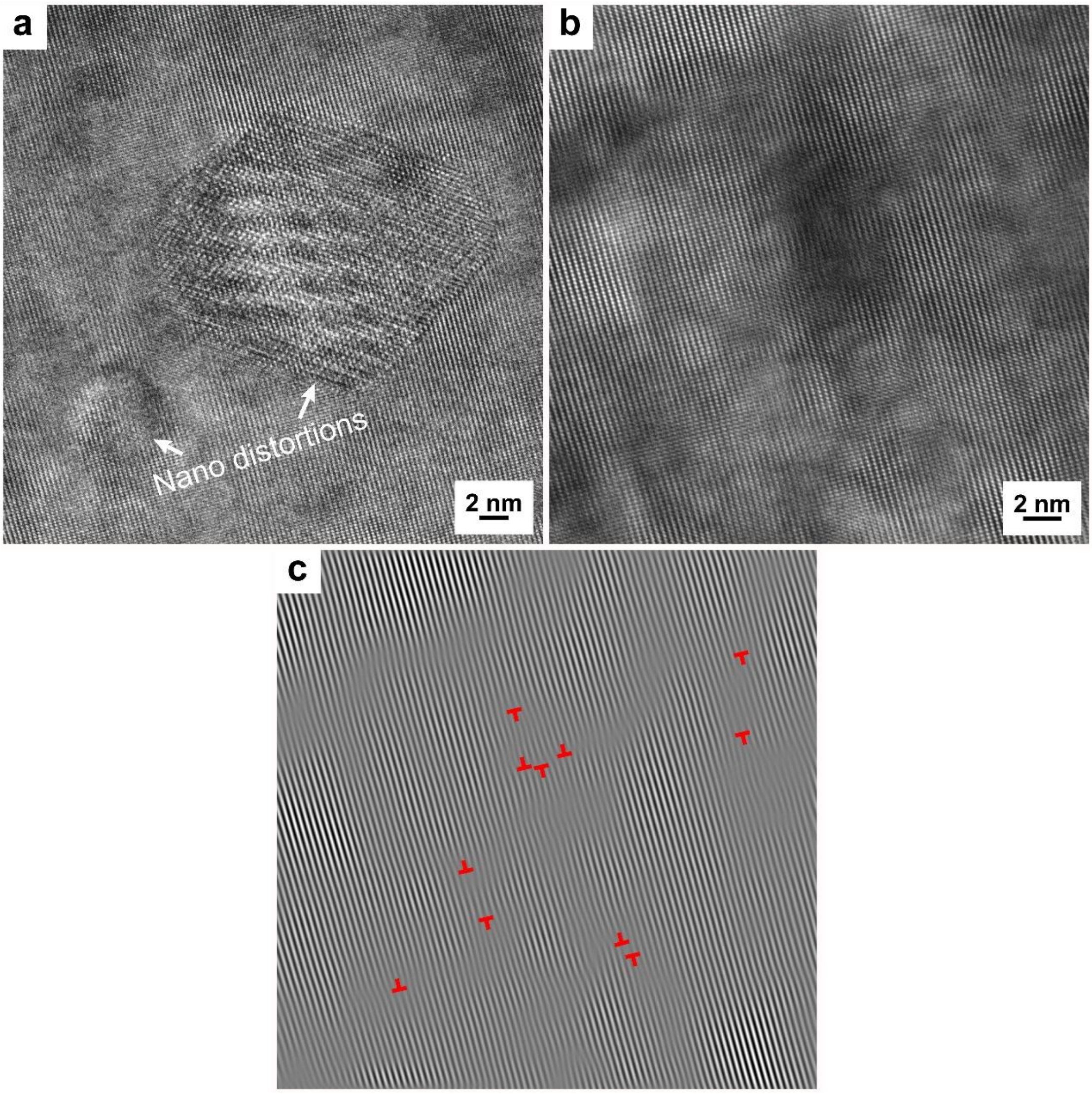

Figure S13. TEM images for the 673A48 sample. The high-resolution TEM (HRTEM) images of **(a)** nano distortions, **(b)** a randomly selected region and **c** the IFFT image of **(b)** in the 673A48 sample.

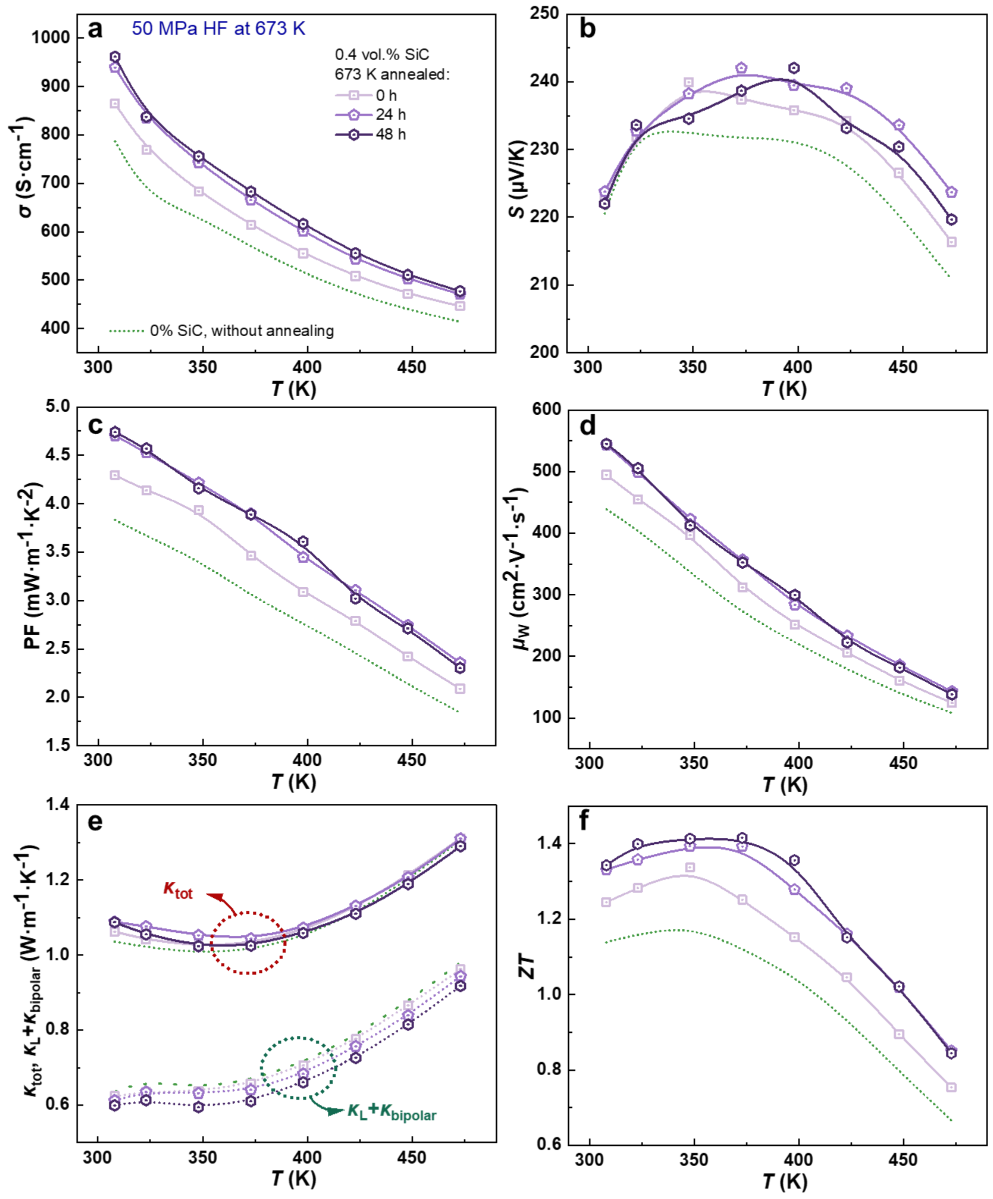

Figure S14. TE performance of for the 0.4 vol% SiC-incorporated $Bi_{0.4}Sb_{1.6}Te_3$ samples. Temperature dependence of **(a)** electrical conductivity, **(b)** Seebeck coefficient, **(c)** power factor, **(d)** weighted mobility, **(e)** total thermal conductivity as well as lattice and bipolar thermal conductivity, and **(f)** *ZT* values for 0.4 vol% SiC-incorporated $Bi_{0.4}Sb_{1.6}Te_3$ samples subjected to annealing with different durations at 673 K followed by HF.

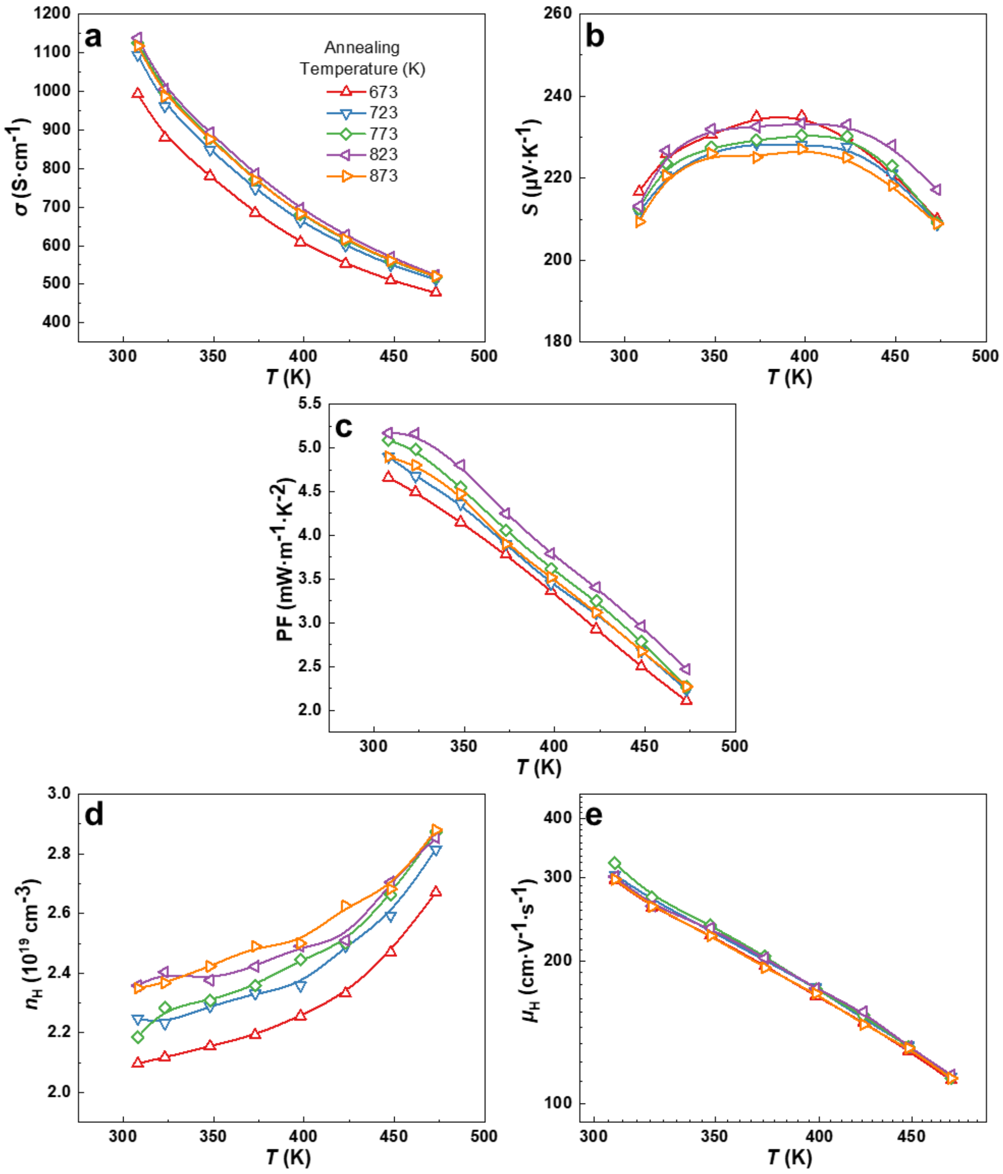

Figure S15. Electrical transport properties of for the 0.4 vol% SiC-incorporated $Bi_{0.4}Sb_{1.6}Te_{3.01}$ samples. Temperature dependence of **(a)** electrical conductivity, **(b)** Seebeck coefficient, **(c)** power factor, **(d)** Hall carrier concentration and **(e)** Hall mobility (inset shows the weighted mobility values at 303 K) for the 0.4 vol% SiC-incorporated $Bi_{0.4}Sb_{1.6}Te_{3.01}$ samples subjected to annealing at different temperatures for 48 h and followed by HF.

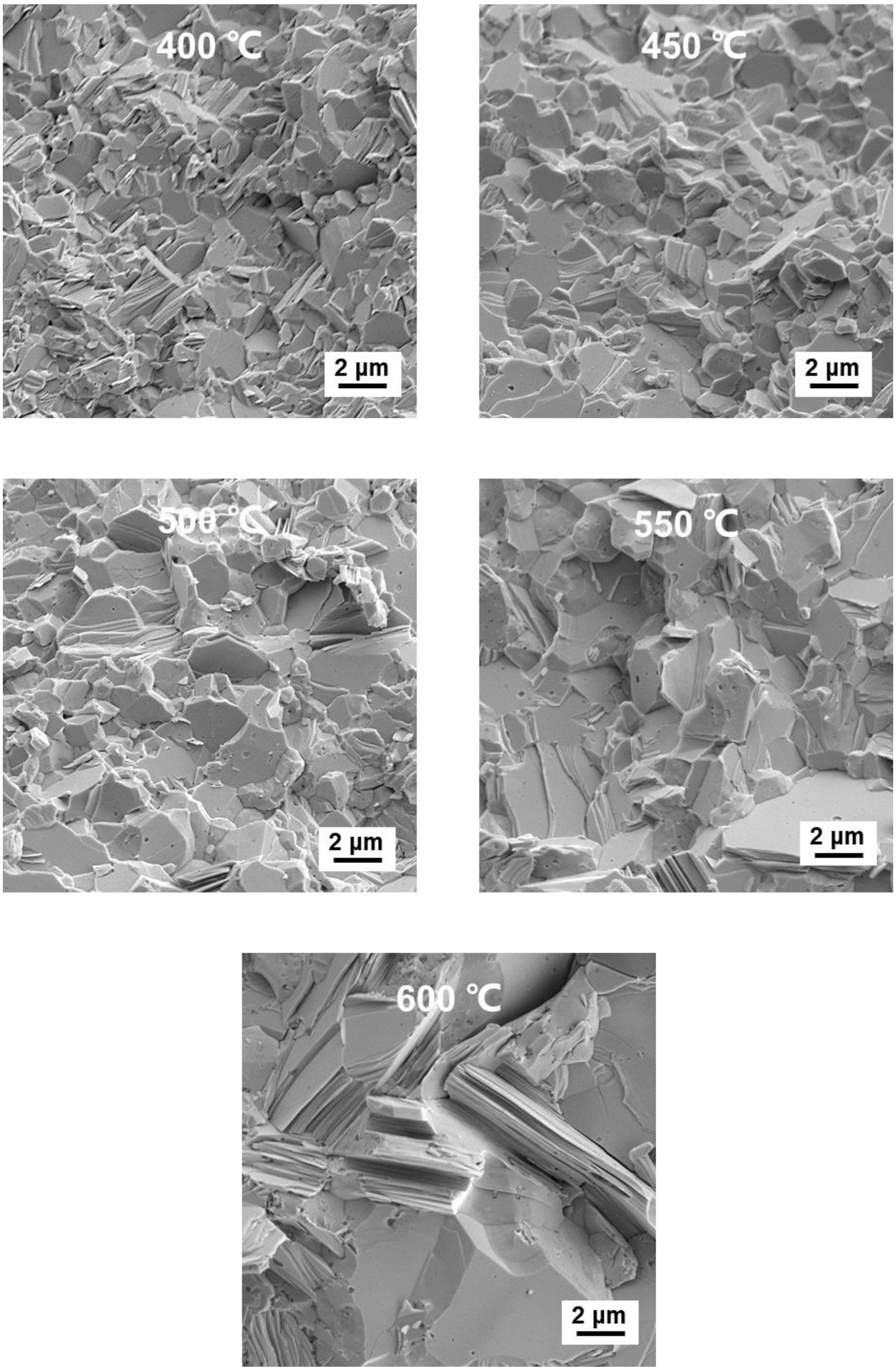

Figure S16. SEM images of the 0.4 vol% SiC-incorporated $Bi_{0.4}Sb_{1.6}Te_{3.01}$ samples. SEM images of the fracture surface for the 0.4 vol% SiC-incorporated $Bi_{0.4}Sb_{1.6}Te_{3.01}$ samples subjected to annealing at different temperatures for 48 h and followed by HF.

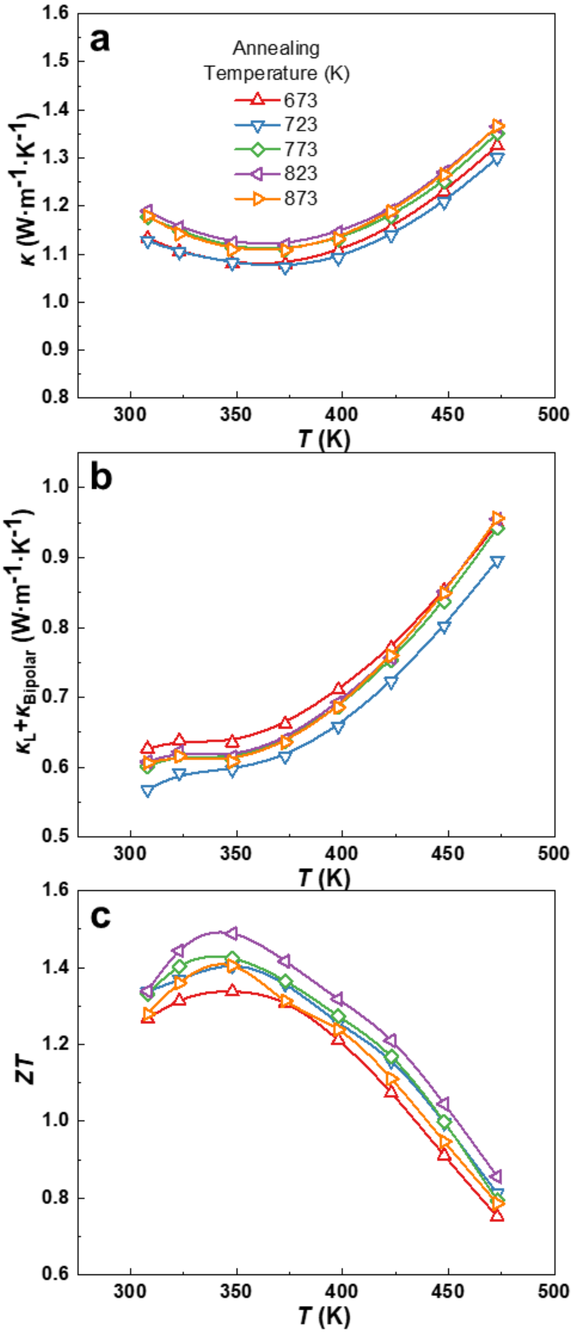

Figure S17. Thermal transport properties and *ZT* values of the 0.4 vol% SiC-incorporated $Bi_{0.4}Sb_{1.6}Te_{3.01}$ samples. Temperature dependence of **(a)** total thermal conductivity, **(b)** lattice and bipolar thermal conductivity, as well as **(c)** *ZT* values for the 0.4 vol% SiC-incorporated $Bi_{0.4}Sb_{1.6}Te_{3.01}$ samples subjected to annealing at different temperatures for 48 h and followed by HF.

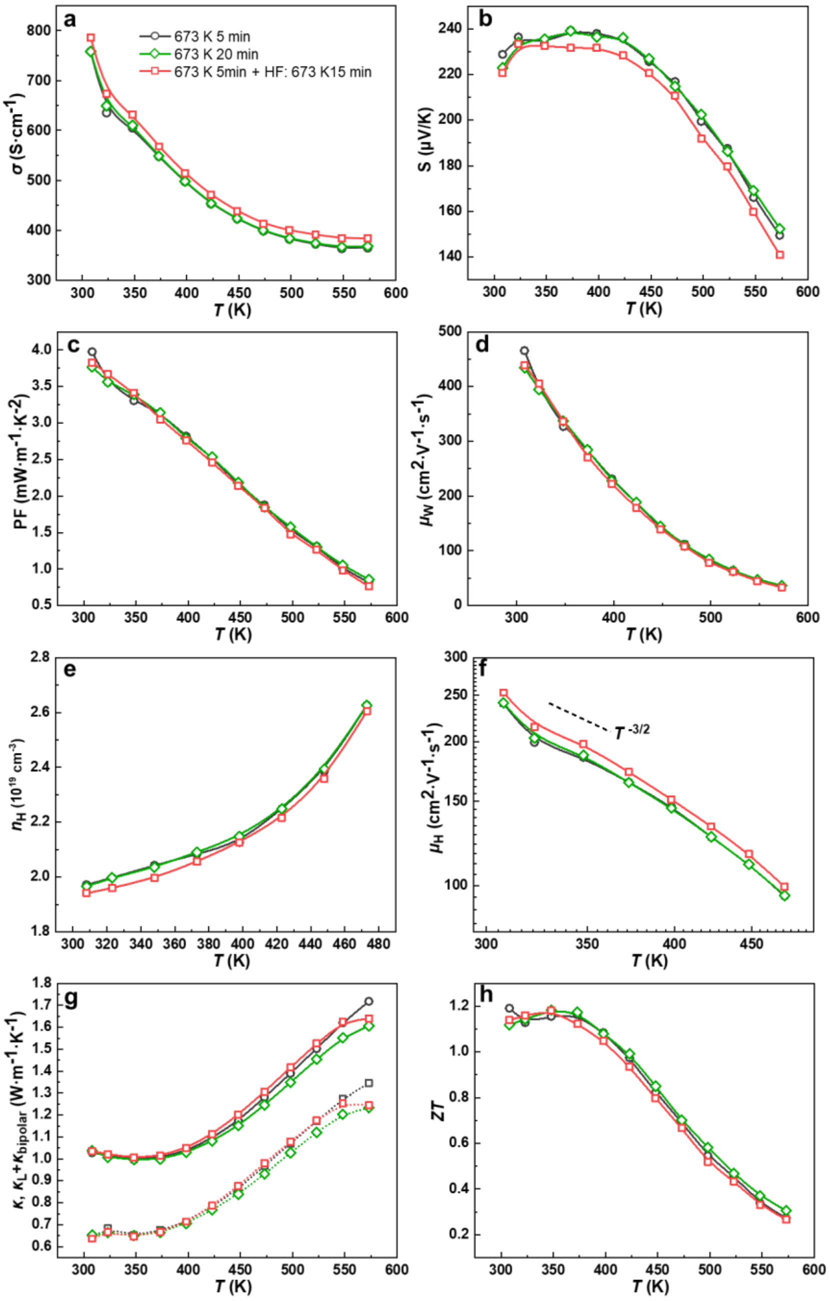

Figure S18. TE performance of the $Bi_{0.4}Sb_{1.6}Te_3$ samples. Temperature dependence of **(a)** electrical conductivity, **(b)** Seebeck coefficient, **(c)** power factor, **(d)** weighted mobility, **(e)** Hall carrier concentration, **(f)** Hall mobility, **(g)** total thermal conductivity and lattice plus bipolar thermal conductivity, and **(h)** *ZT* values for $Bi_{0.4}Sb_{1.6}Te_3$ samples prepared under different conditions without annealing.

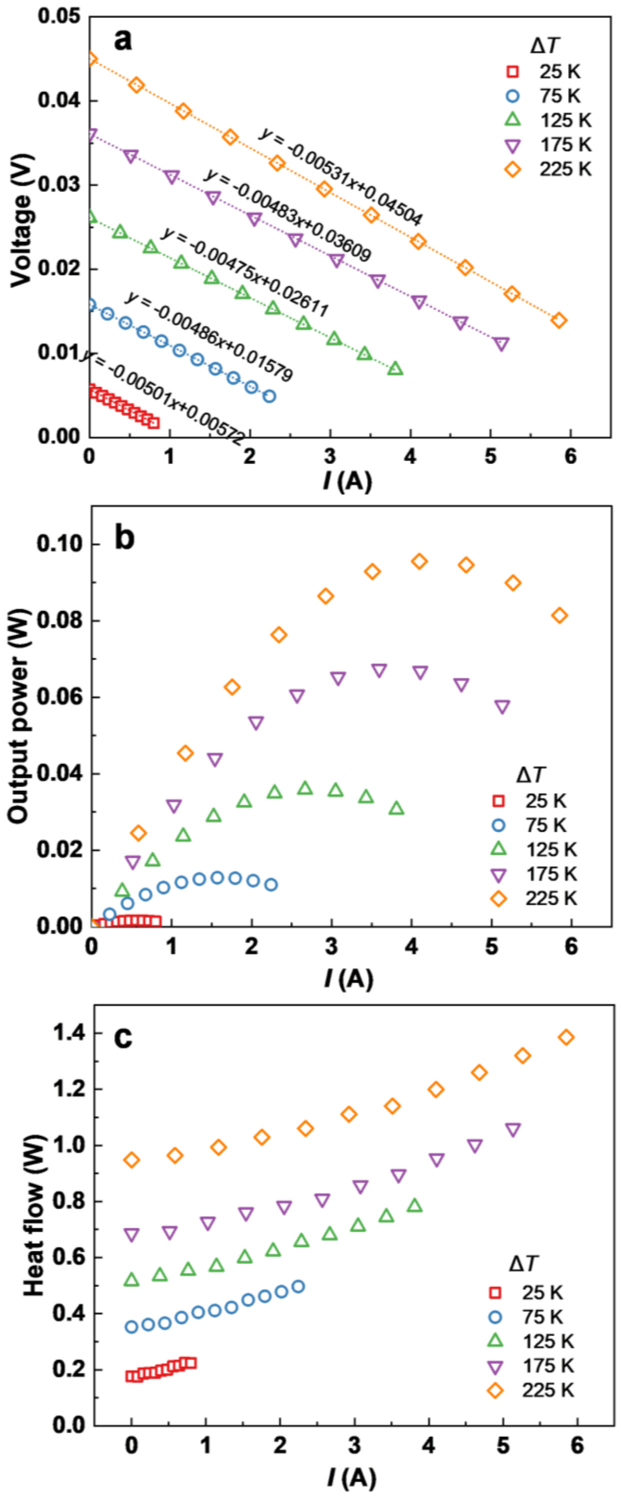

Figure S19. Measured results for the single-leg device. The measured **(a)** output voltage, **(b)** output power and **(c)** heat flow for the single-leg device prepared using the 823A48ST sample.

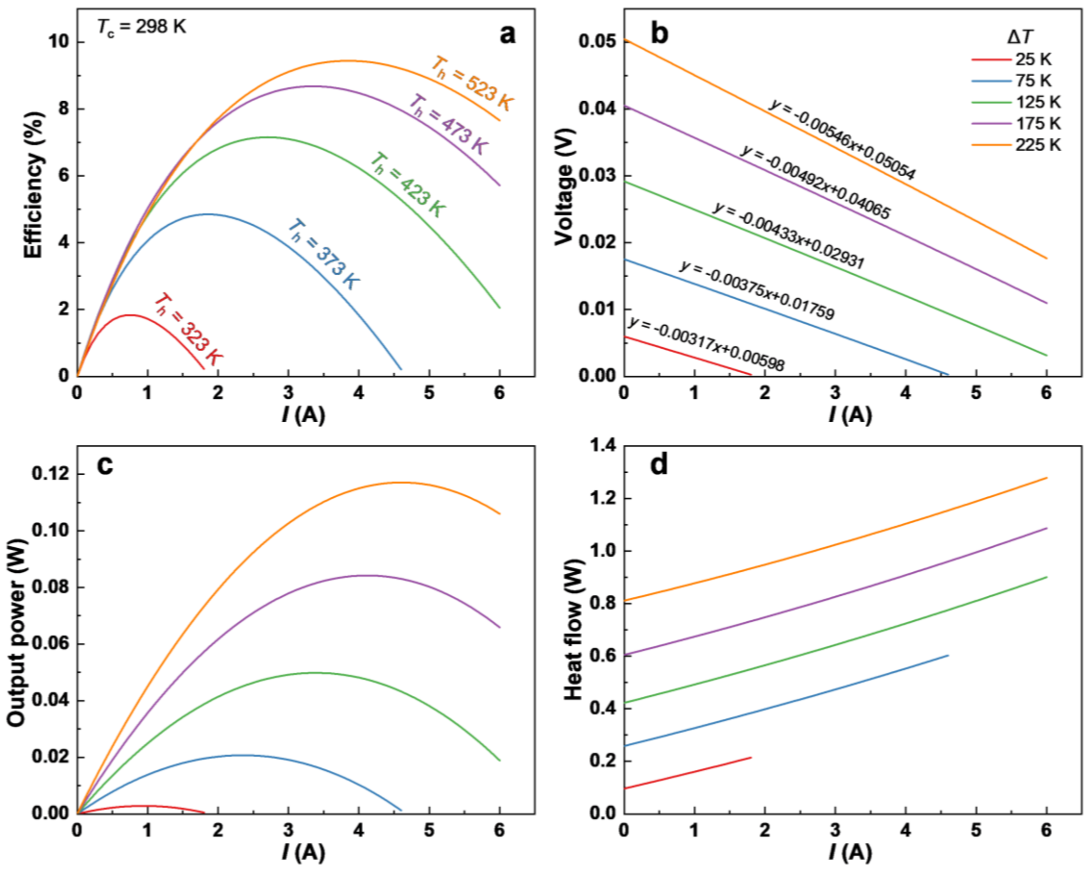

Figure S20. Simulated results for the single-leg device. The theoretically predicted **(a)** TE conversion efficiency, **(b)** output voltage, **(c)** output power and **(d)** heat flow for the single-leg device prepared using the 823A48ST sample.

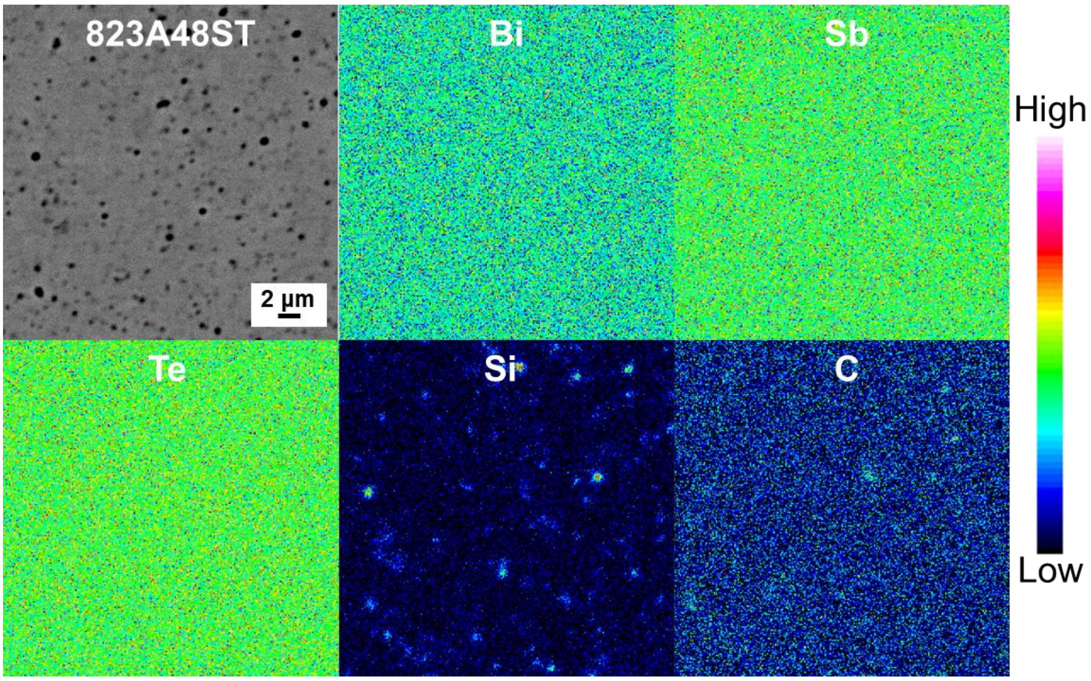

Figure S21. The EPMA mapping for Sample 4. The EPMA mapping for elements of Bi, Sb, Te, Si and C on the polished surface of the 823A48ST sample.

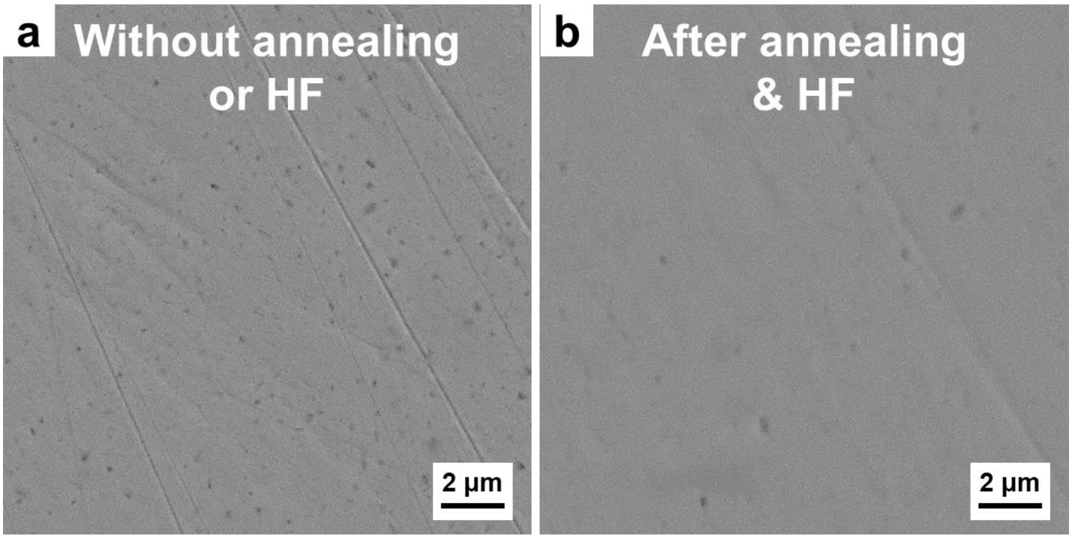

Figure S22. SEM images of the $Bi_{0.4}Sb_{1.6}Te_3$ samples. The SEM images of the polished surface for the $Bi_{0.4}Sb_{1.6}Te_3$ samples **(a)** without annealing or HF and **(b)** subjected to A-HF process.

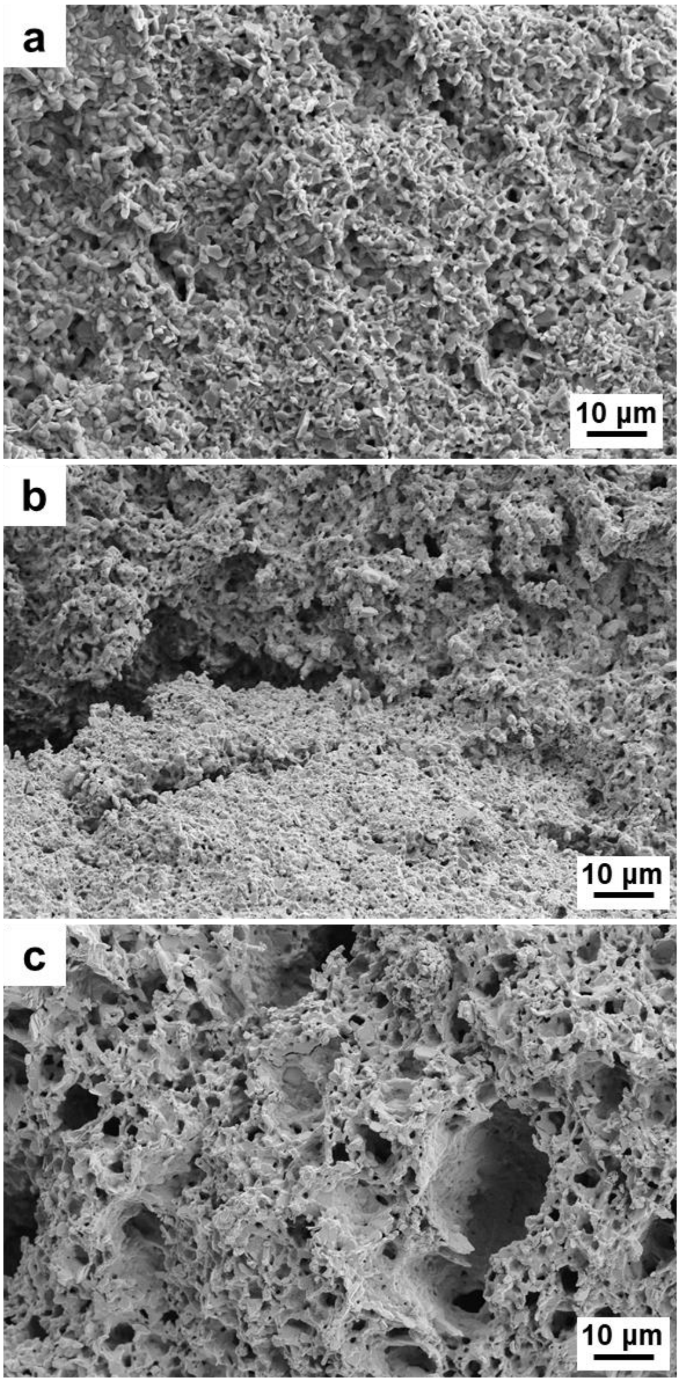

Figure S23. SEM images of the annealed $(Bi,Sb)_2Te_3$-based samples. SEM images of fracture surfaces for the samples annealed without HF: **(a)** the $Bi_{0.4}Sb_{1.6}Te_3$ annealed at 673 K for 48 h, **(b)** the 0.4 vol% SiC-incorporated $Bi_{0.4}Sb_{1.6}Te_{3.01}$ samples annealed at 673 K for 48 h and **(c)** the 0.4 vol% SiC-incorporated $Bi_{0.4}Sb_{1.6}Te_{3.01}$ samples annealed at 823 K for 48 h.

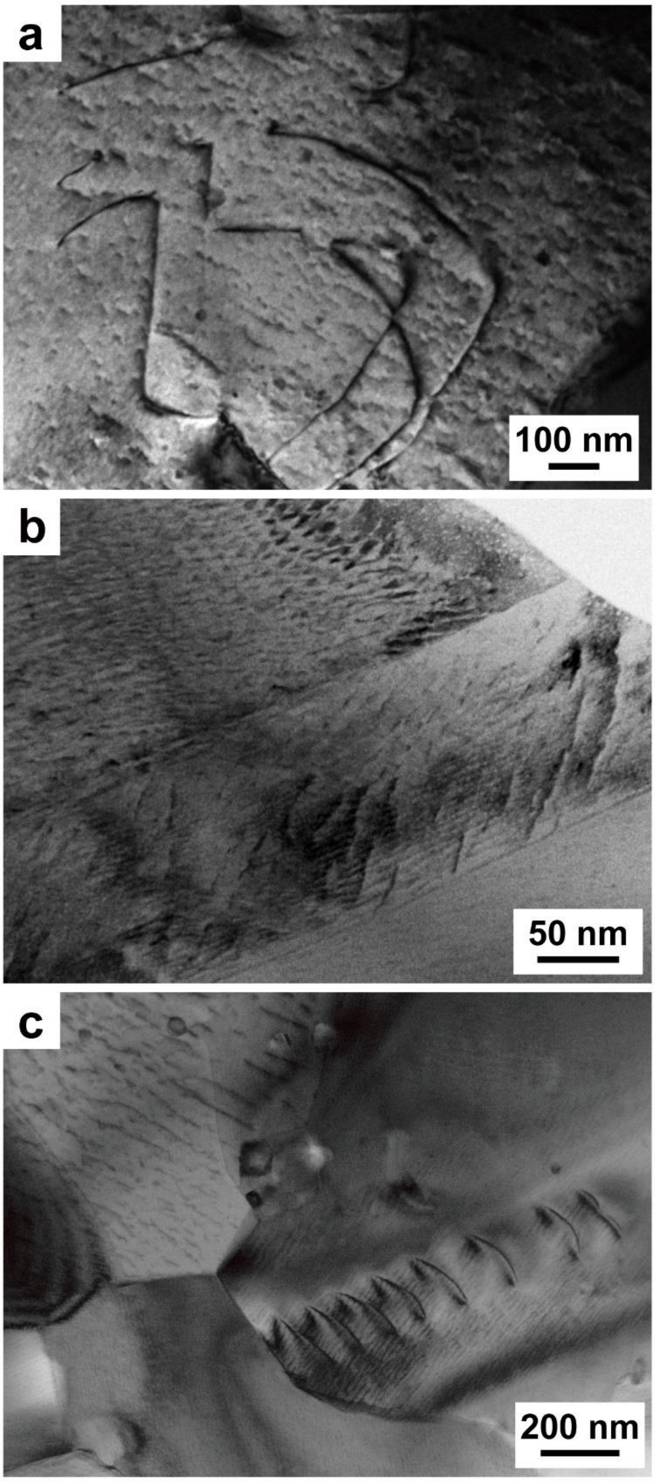

Figure S24. TEM images of the dislocations in Sample 4. (a)-(c) The low-magnification TEM images for the dislocations with different morphologies.

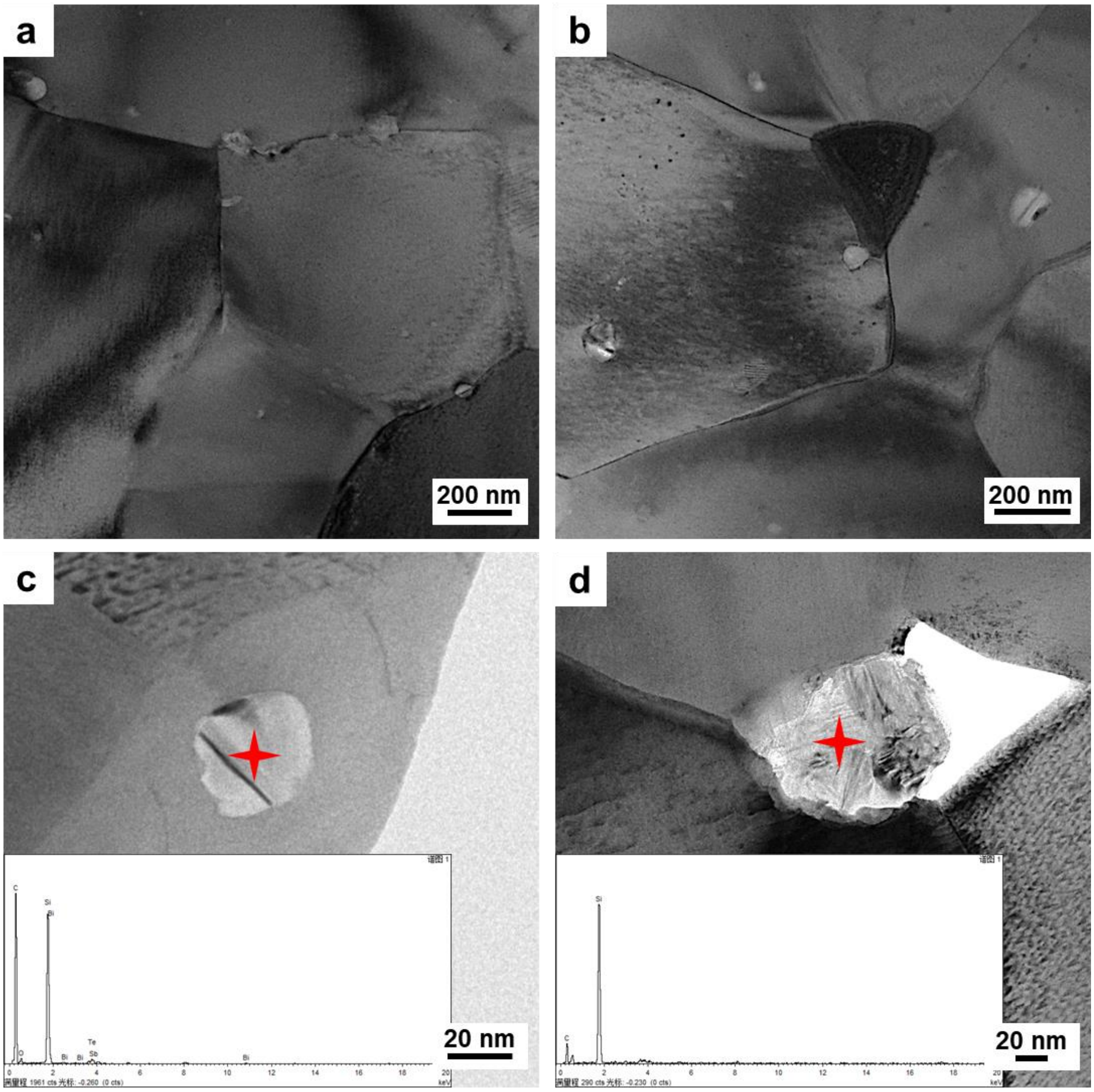

Figure S25. TEM images of the nano SiC in Sample 4. (a),(b) Low-magnification TEM images for randomly selected regions in the 823A48ST sample. Plentiful nanoparticles were found incorporated in the sample. **(c),(d)** The enlarged view of the incorporated nanoparticles by low-magnification TEM, which was recognized as nano SiC by EDS point analysis.

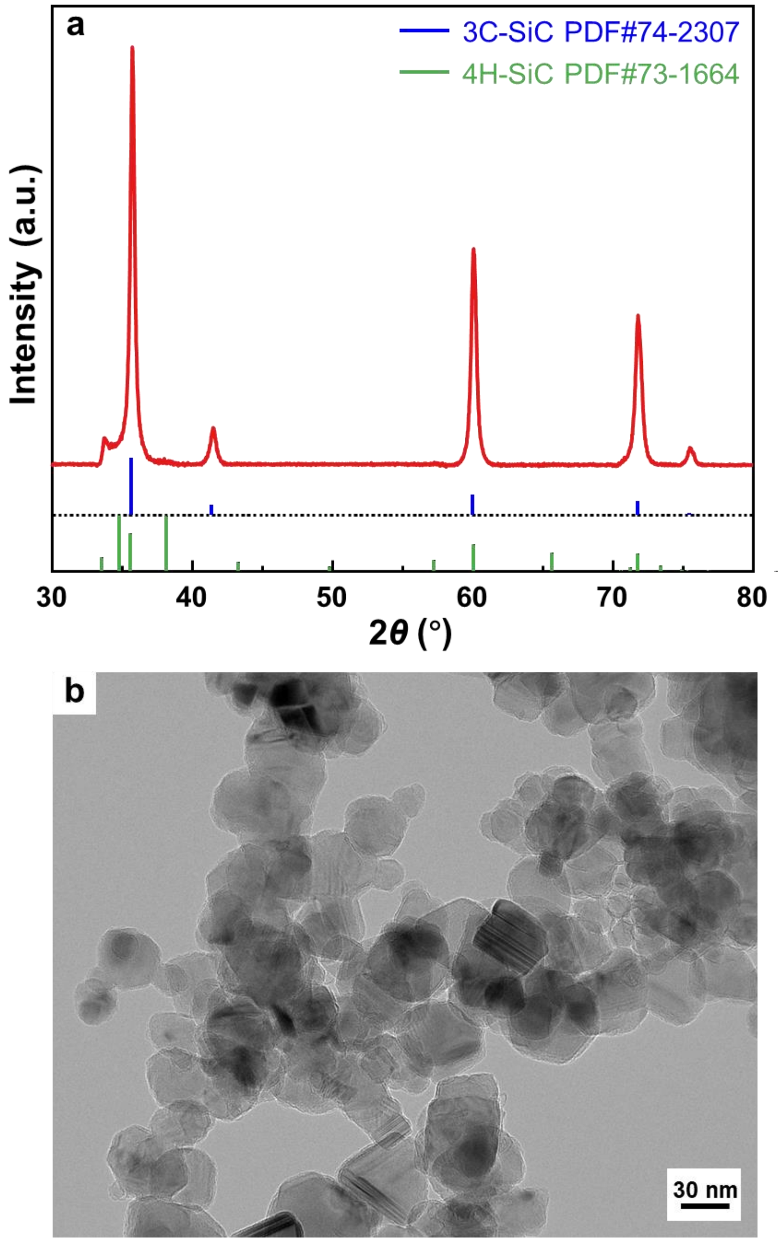

Figure S26. Characterization of the SiC nanoparticles. The **(a)** XRD pattern and **(b)** TEM image of the SiC nanoparticles.

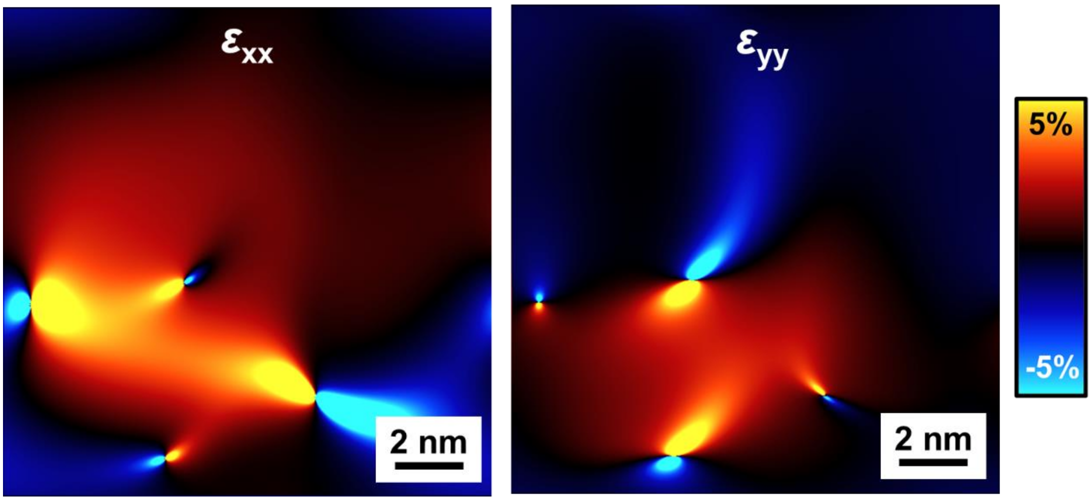

Figure S27. GPA of a selected region in Sample 4. The mapping of normal strains for area F in Fig. 4e obtained by GPA.

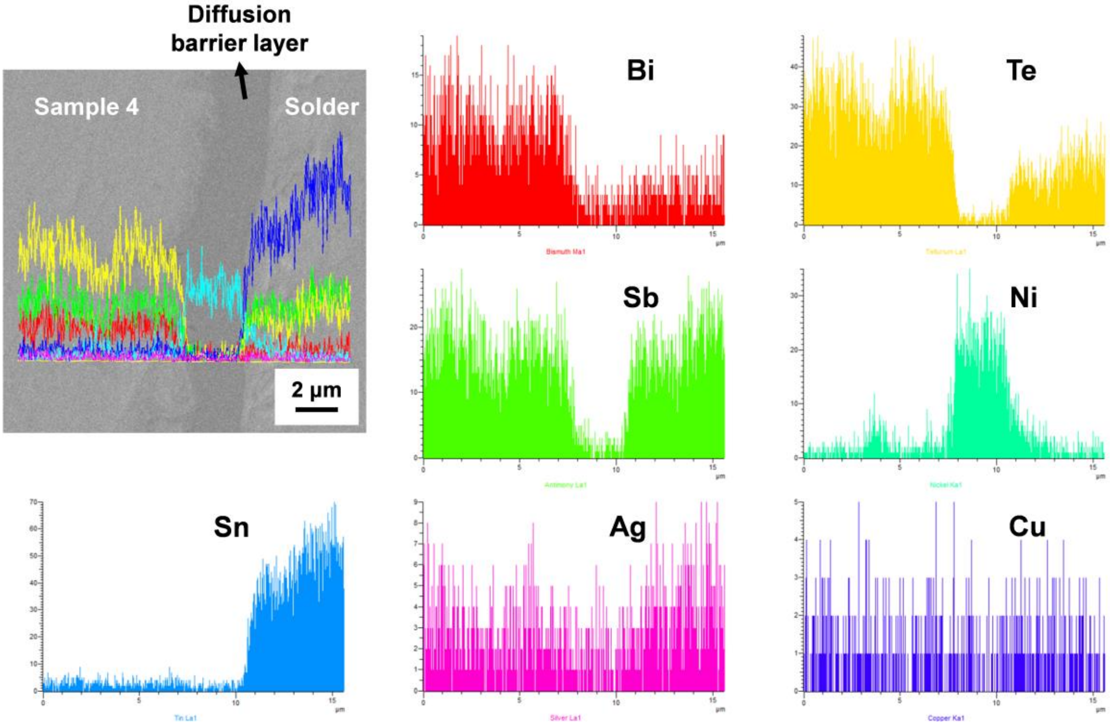

Figure S28. The SEM image and the EDS line scanning of the interface between Sample 4, the diffusion barrier layer and the solder

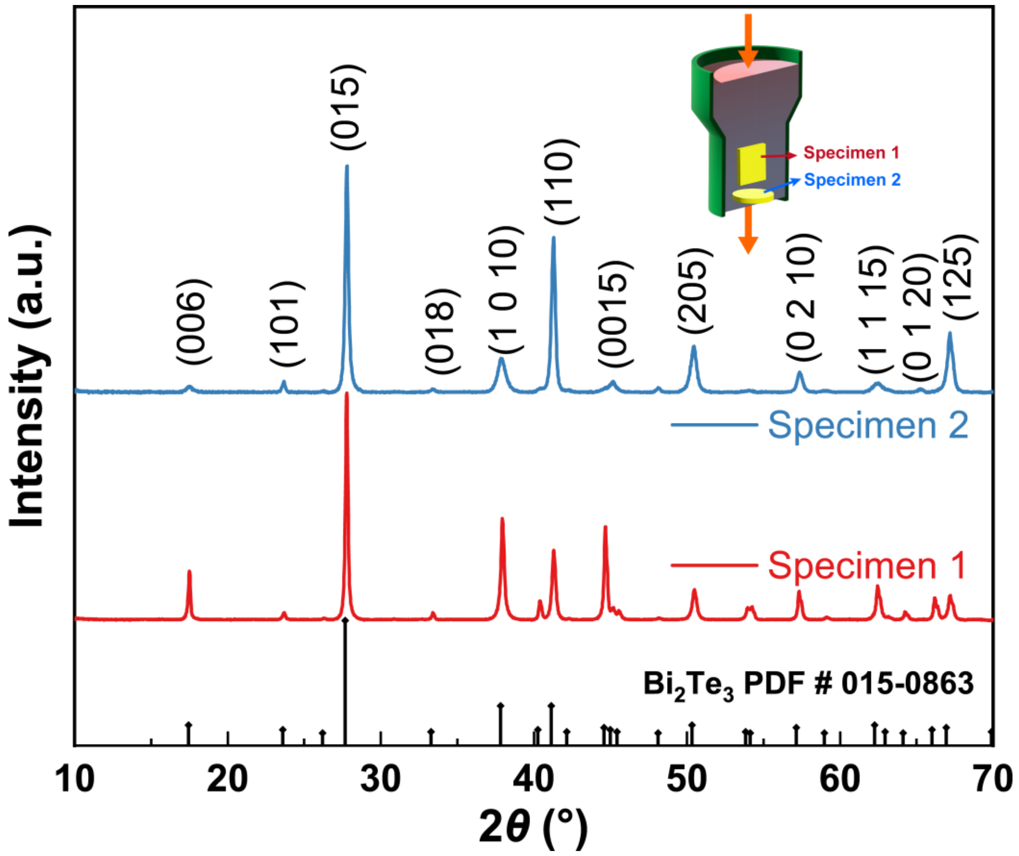

Figure S29. XRD pattern of the n-type Bi_2Te_3 used in micro-PCs. The two specimens represent measurements taken along the directions perpendicular and parallel to the extrusion direction, respectively.

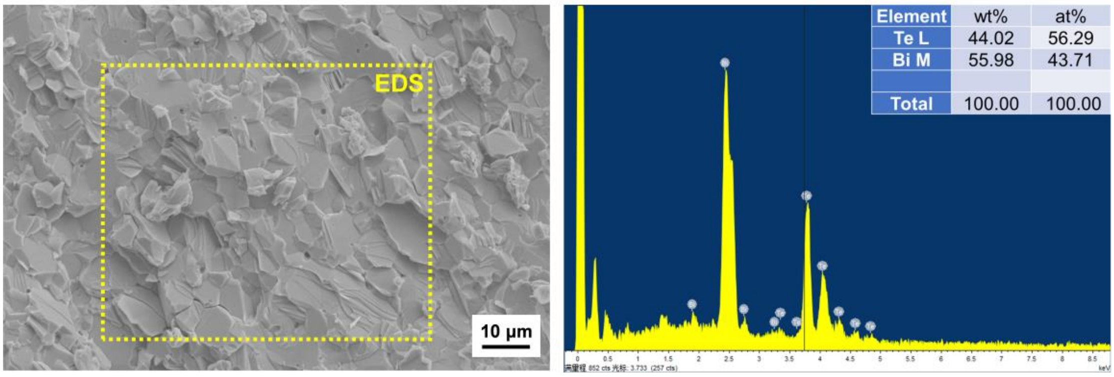

Figure S30. SEM image of the fracture surface of the n-type Bi_2Te_3 used in micro PCs, along with the EDS composition analysis result of the selected region.

Table S1. Density of the samples in this work.

Annealing duration (h)	Annealing temperature (K)	HF	Nano-SiC	Excess Te	Density (g/cm^3)	Relative density
-	-	×	×	×	6.626	97.5%
-	-	√	×	×	6.451	95.0%
6	673	√	×	×	6.670	98.2%
12	673	√	×	×	6.681	98.3%
24	673	√	×	×	6.678	98.3%
48	673	√	×	×	6.678	98.3%
-	-	√	√	×	6.568	96.7%
24	673	√	√	×	6.644	97.8%
48	673	√	√	×	6.650	97.9%
48	673	√	√	√	6.651	97.9%
48	723	√	√	√	6.661	98.1%
48	773	√	√	√	6.666	98.1%
48	823	√	√	√	6.674	98.2%
48	873	√	√	√	6.674	98.2%

References

1. Cai B, Zhuang H-L, Cao Q *et al.* Practical high-performance $(Bi,Sb)_2Te_3$-based thermoelectric nanocomposites fabricated by nanoparticle mixing and scrap recycling. *ACS Appl Mater Interfaces* 2020; **12**: 16426–35.

2. Li J, Tan Q, Li J-F *et al.* BiSbTe-based nanocomposites with high *ZT*: The effect of SiC nanodispersion on thermoelectric properties. *Adv Funct Mater* 2013; **23**: 4317–23.

3. Kim H-S, Gibbs ZM, Tang Y *et al.* Characterization of Lorenz number with Seebeck coefficient measurement. *APL Mater* 2015; **3**: 041506.

4. Zhuang H-L, Pei J, Cai B *et al.* Thermoelectric performance enhancement in BiSbTe alloy by microstructure modulation via cyclic spark plasma sintering with liquid phase. *Adv Funct Mater* 2021; **31**: 2009681.

5. Chetty R, Babu J, Mori T. Best practices for evaluating the performance of thermoelectric devices. *Joule* 2024; **8**: 556–62.

6. Gromov G, Kondratiev D, Rogov A *et al.* Z-meter: Easy-to-use Application and Theory. In: *Proc. Of VI Eur. Workshop on Thermoelectrics, Freiburg*, 2001.

7. Harman TC, Cahn J, Logan M. Measurement of thermal conductivity by utilization of the Peltier effect. *J Appl Phys* 1959; **30**: 1351–9.

8. Qin B, Wang D, Liu X *et al.* Power generation and thermoelectric cooling enabled by momentum and energy multiband alignments. *Science* 2021; **373**: 556–61.

9. Liu D, Wang D, Hong T *et al.* Lattice plainification advances highly effective SnSe crystalline thermoelectrics. *Science* 2023; **380**: 841–6.

10. Qin B, Wang D, Hong T *et al.* High thermoelectric efficiency realized in SnSe crystals via structural modulation. *Nat Commun* 2023; **14**: 1366.

11. Zhu TJ, Hu LP, Zhao XB *et al.* New insights into intrinsic point defects in V_2VI_3 thermoelectric materials. *Adv Sci* 2016; **3**: 1600004.

12. Cutler M, Mott NF. Observation of Anderson localization in an electron gas. *Phys Rev* 1969; **181**: 1336.

13. Rowe DM. *Thermoelectrics Handbook: Macro to Nano*. CRC Press, 2018.

14. Rowe DM. *CRC Handbook of Thermoelectrics*. Boca Raton: CRC Press, 1995.

15. Snyder GJ, Snyder AH, Wood M *et al.* Weighted mobility. *Adv Mater* 2020; **32**: 2001537.

16. Lotgering FK. Topotactical reactions with ferrimagnetic oxides having hexagonal crystal structures—I. *J Inorg Nucl Chem* 1959; **9**: 113–23.

17. Zhang X, Bu Z, Shi X *et al.* Electronic quality factor for thermoelectrics. *Sci Adv* 2020; **6**: eabc0726.
